# Seasonal Changes in the Oxidative Potential of Urban Air Pollutants: The Influence of Emission Sources and Proton- and Ligand-Mediated Dissolution of Transition Metals

**DOI:** 10.1021/acsestair.4c00093

**Published:** 2024-08-29

**Authors:** Pourya Shahpoury, Steven Lelieveld, Deepchandra Srivastava, Andrea Baccarini, Jacob Mastin, Thomas Berkemeier, Valbona Celo, Ewa Dabek-Zlotorzynska, Tom Harner, Gerhard Lammel, Athanasios Nenes

**Affiliations:** † Environmental and Life Sciences, Trent University, Peterborough K9L0G2, Canada; ‡ Multiphase Chemistry Department, Max Planck Institute for Chemistry, Mainz 55128, Germany; § Division of Environmental Health and Risk Management, School of Geography, Earth & Environmental Sciences, University of Birmingham, Edgbaston, Birmingham B152TT, United Kingdom; ∥ Laboratory of Atmospheric Processes and their Impacts, School of Architecture, Civil and Environmental Engineering, École Polytechnique Fédérale de Lausanne, Lausanne CH-1015, Switzerland; ⊥ Air Quality Processes Research Section, Environment and Climate Change Canada, Toronto M3H5T4, Canada; # Analysis and Air Quality Section, Environment and Climate Change Canada, Ottawa K1V1C7, Canada; ∇ Institute of Chemical Engineering Sciences, Foundation for Research and Technology Hellas, Patras GR-26504, Greece

**Keywords:** health effects, oxidative stress, reactive oxygen species, ozone, nitrogen dioxide, fine particulate matter, secondary organic matter

## Abstract

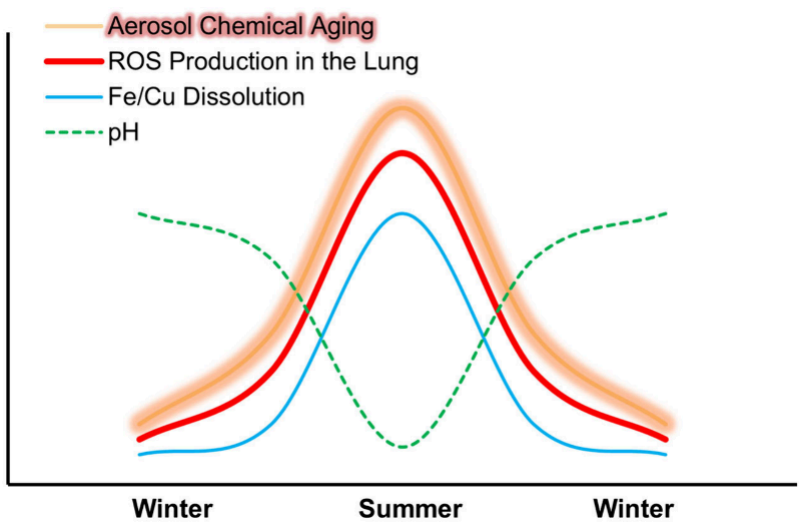

The inhalation of fine particulate matter (PM_2.5_) is a major contributor to adverse health effects from air pollution worldwide. An important toxicity pathway is thought to follow oxidative stress from the formation of exogenous reactive oxygen species (ROS) in the body, a proxy of which is oxidative potential (OP). As redox-active transition metals and organic species are important drivers of OP in urban environments, we investigate how seasonal changes in emission sources, aerosol chemical composition, acidity, and metal dissolution influence OP dynamics. Using a kinetic model of the lung redox chemistry, we predicted ROS (O_2_^•–^, H_2_O_2_, ^•^OH) formation with input parameters comprising the ambient concentrations of PM_2.5_, water-soluble Fe and Cu, secondary organic matter, nitrogen dioxide, and ozone across two years and two urban sites in Canada. Particulate species were the largest contributors to ROS production. Soluble Fe and Cu had their highest and lowest values in summer and winter, and changes in Fe solubility were closely linked to seasonal variations in chemical aging, the acidity of aerosol, and organic ligand levels. The results indicate three conditions that influence OP across various seasons: (a) low aerosol pH and high organic ligand levels leading to the highest OP in summer, (b) opposite trends leading to the lowest OP in winter, and (c) intermediate conditions corresponding to moderate OP in spring and fall. This study highlights how atmospheric chemical aging modifies the oxidative burden of urban air pollutants, resulting in a seasonal cycle with a potential effect on population health.

## Abstract 2

Using field measurements and model simulations, this work investigates if seasonal changes in emission sources, aerosol acidity and composition, and metal dissolution influence the oxidative potential of urban air.

## 1 Introduction

Air pollution can lead to adverse health effects within populations, including respiratory, cardiovascular, and neurological diseases and premature death.^1−9^ The health effects have been associated with the inhalation of fine particulate matter (PM) with an aerodynamic diameter of ≤2.5 μm, i.e., PM_2.5_. Hence, the PM_2.5_ mass concentration is commonly used as a metric to assess air quality. Mounting evidence suggests that a key toxicological pathway follows the initiation of oxidative stress due to the formation of exogenous reactive oxygen species (ROS) in the body.^5,10−19^ Therefore, more recently, the oxidative potential (OP) has been adopted as a complementary metric to the PM_2.5_ mass concentration in the study of air pollution health effects.^12,20^ ROS are formed following the reaction of redox-active pollutants, such as transition metals and quinones, in the epithelial lining fluid (ELF). This process involves the transfer of electrons from reduced metals or quinones to molecular oxygen to form superoxide anions (O_2_^•–^), hydrogen peroxide (H_2_O_2_), and subsequently hydroxyl radicals (^•^OH) via Fenton-type reactions. The excess formation of ROS can lead to the oxidation of cellular components, such as lipids and proteins. ROS formation can be quantified using a variety of acellular and cellular assays or model simulation.^20−31^ The KM-SUB-ELF kinetic model has been used previously to study ROS formation from the reaction of PM-bound transition metals, organic matter, and reactive gases in a simulated ELF, including epidemiological aspects, dependency on particle size distribution, and intracellular formation of ROS.^32−39^ The model uses the ambient concentrations of water-soluble trace metals Fe and Cu, secondary organic matter (SOM), ozone (O_3_), and nitrogen dioxide (NO_2_) to simulate the chemical reactions that lead to the formation of ROS. In a companion paper, we compared the ^•^OH formation obtained using the kinetic model with that from an acellular assay and found similar spatial trends across various urban sites in Canada (Spearman correlation *r*_s_ ≥ 0.73).^39^ The model-predicted ^•^OH was closely associated with the concentration of water-soluble Fe, aerosol pH, and levels of oxalate (an abundant organic ligand in ambient PM). The abundance of water-soluble metals in PM depends on various factors including the type of emission sources, particle size, and proton- and ligand-mediated dissolution processes.^40−48^ Aerosol pH and oxalate are known to have seasonal cycles, with aerosol becoming more acidic and oxalate levels increasing during the summer months.^46,49,50^ Such temporal variations can influence the levels of water-soluble metals and, consequently, the capacity of PM to generate ROS in the lung across various seasons. To the best of our knowledge, no previous study has explored this aspect, and particularly not using the kinetic model. In this work, we investigated this topic using an extensive chemical characterization of the aerosol gas and particulate phases at two urban sites for two consecutive years under the Canadian National Air Pollution Surveillance (NAPS) program. The specific aims of the study were (a) to identify the emission sources that contribute to water-soluble Fe and Cu in various seasons, (b) to explore the seasonal links between aerosol (particulate phase) acidity, oxalate concentrations, and the solubility of Fe and Cu in PM_2.5_, and (c) to investigate if seasonal changes in aerosol chemical composition and acidity correspond to changes in ROS production in the lung.

## 2 Methodology

### 2.1 Study Locations

Ambient air was collected from NAPS sites in Toronto (*n* = 243) and Hamilton (*n* = 243; Table 1) in 2017 and 2018. These sites were previously characterized in terms of emission sources.^46,51−53^ While both sites are classified as large urban areas, the site in Toronto is considered a near-road site and is primarily influenced by transportation emissions (situated ∼10 m from one of North America’s busiest highways). In contrast, the Hamilton site is influenced primarily by industrial activities and, in particular, metal manufacturing (situated ∼3 km from the industrial complex). Moreover, the Toronto site is classified under the NAPS scheme as highly populated (≥150 000 living within 4 km of the study site), whereas Hamilton is in the midpopulation range (Table 1).

**Table 1:** Sampling Site Information

	NAPS ID	coordinates	source type	number of samples	PM_2.5_ concentration min–max (μg m^–3^)^c^
Toronto^a^	60438	43.71111, −79.54340	T, LU, P6, C	243	2.43–30.4
Hamilton^b^	60512	43.25790, −79.86154	PS, LU, P5, R	243	1.39–26.8

a Near-road site.

b Industrial site. Emission source type (T: transportation influence; PS: point-source influence); urbanization (LU: large urban area); neighborhood population residing within 4 km of the site (P5: 100 000–149 999; P6: ≥150 000); local land use (C: commercial, R: residential).

c 24 h mean values.

### 2.2 Sampling and Chemical Analyses

The details of the sampling and analytical procedure used here have been discussed in previous publications.^39,46,51,54,55^ Briefly, air samples were collected at each site using (a) a Dichotomous sampler (flow rate: 15 L min^–1^; Partisol, 2000i-D, Thermo Scientific, Waltham, U.S.) and (b) a SUPER SASS-Plus sequential speciation sampler (flow rate: 10 L min^–1^; Met One Instruments, Inc., Grants Pass, U.S.); 24 h samples were collected at each site once every 3 days, resulting in the sampled air volumes of 21.6 and 14.4 m^3^ with the Dichotomous (at PM_2.5_ channel) and SUPER SASS-Plus samplers, respectively. The Dichotomous samplers were mounted with polytetrafluoroethylene (PTFE; 47 mm i.d.) membranes, and the PM_2.5_ samples from these samplers were analyzed for the near-total concentrations of trace metals, including Fe and Cu, using inductively coupled plasma mass spectrometry (ICP-MS) following microwave-assisted acid digestion.^56^ The SUPER SASS-Plus samplers were equipped with PM_2.5_ inlets and ChemComb cartridges mounted with quartz fiber filters (QFF; 47 mm i.d.), PTFE membranes (47 mm i.d.), and denuders and were used for various chemical analyses. The PTFE membrane downstream of the denuders was extracted using deionized water and analyzed using ion chromatography (IC) for water-soluble anions and cations as well as organic acids, including oxalate, sulfate (SO_4_^2–^), nitrate (NO_3_^–^), ammonium (NH_4_^+^), sodium, calcium, magnesium, and potassium. The aqueous extracts were also analyzed using ICP-MS for water-soluble trace metals including Fe and Cu. Additionally, 1 cm^2^ punches of QFF were analyzed for organic carbon (OC) and elemental carbon (EC) using the IMPROVE protocol, whereas denuders were extracted with water and analyzed for gaseous HNO_3_, SO_2_, and NH_3_ using IC. The limits of quantification (LOQs) were determined using mean + three standard deviations of the concentrations in blanks (*n* = 12). Where the analyte concentrations in samples exceeded the LOQs, the mean blank concentrations were subtracted from the sample concentrations. The values below the LOQs were not included in the subsequent data analysis. Moreover, the hourly concentrations of ground-level O_3_ and NO_2_ at the sites were obtained from the NAPS database for the duration of the study, and 24 h mean values were calculated for these measurements.

### 2.3 Source Apportionment Method

The U.S. Environmental Protection Agency’s positive matrix factorization (PMF) model (version 5.0) was used for source apportionment.^57,58^ The analysis focused mainly on identifying the sources of water-soluble Fe and Cu in PM_2.5_, due to the use of these elements as input parameters in the KM-SUB-ELF model (see section 2.4). The chemical species were selected based on several criteria: (a) the species were strong markers and showed distinct source signatures across urban and background sites in previous studies within the NAPS program.^46,51,52,59−62^ (b) The species could be linked to potential sources of water-soluble Fe and Cu. (c) The species were present in >60% of the samples. These included EC, OC, oxalate, SO_4_^2–^, NO_3_^–^, NH_4_^+^, levoglucosan, Si, Ca, Mn, Zn, Sn, Ba, Pb, Cd, and water-soluble Fe and Cu. The near-total concentrations of Fe and Cu were not included in the analysis, as this prevented the model from converging. Outliers were identified using a single iteration of Grubbs’ test, and the concentrations that were found to be outliers were not included in the analysis. EC and OC are typically related to primary combustion sources (e.g., tailpipe emission, wood burning).^63^ Oxalate is associated with photochemically aged aerosol and often correlates with SO_4_^2–^.^50,64^ Moreover, SO_4_^2–^, NO_3_^–^, and NH_4_^+^ are related to secondary sources, whereas nitrogen oxides (NO_*x*_) and ammonia (NH_3_), the precursors for NO_3_ and NH_4_, are emitted from various primary sources.^49,65−67^ Cu, Sn, and Zn are related to the breakdown of motor vehicle brake pads and lubricants, and Fe and Mn are associated with primary traffic emissions (abrasion of brake pads) or crustal elements.^51,55^ The clustering of Fe, Mn, and Zn has also been associated with metallurgical (ferrous smelting) emissions, while Pb and Cd are related to high-temperature industrial emissions, such as coal combustion or coking emissions.^51,52,61^ Ba is typically associated with nonexhaust traffic emissions such as brake wear, whereas Si and Ca are related to resuspended dust from crustal matter. Levoglucosan is a specific tracer of biomass burning.^68−70^

The selection of input parameters for the PMF analysis followed the criteria established in previous studies.^71−73^ Determining the optimal PMF solution involved a thorough examination of factor chemical profiles, temporal trends, correlations with external tracers, bootstrap runs to assess solution stability, and comparisons between the modeled and measured data. To directly assess source contributions to daily mass concentrations, the concentration of PM_2.5_ was included as a total variable in the model (with large uncertainty).

### 2.4 Modeling of Oxidative Potential

The OP of PM_2.5_ was modeled using KM-SUB-ELF, which consists of three compartments, i.e., the lung gas phase, the ELF surfactant layer, and the bulk ELF.^37,38^ The ELF surfactant layer at the gas–liquid interface contains lipids and proteins (1-palmitoyl-2-oleoylglycerol, surfactant protein B). The bulk ELF consists of five aqueous layers containing antioxidants (ascorbic acid, glutathione, uric acid, and α-tocopherol) and antioxidant enzymes (superoxide dismutase, catalase). The model simulates the air exchange in the lung, adsorption and desorption of gases to and from the surfactant layer, chemical transport between the surfactant layer and bulk ELF, diffusion within the bulk ELF, and chemical reactions in the gas phase and the bulk ELF (including 23 reactions in the gas phase, six reactions in the surfactant layer, and 96 reactions in the bulk ELF). In KM-SUB-ELF, the production of ROS from transition metals and quinones in aqueous solution is based on kinetic data from Charrier et al.^74^ and Charrier and Anastasio.^75^ The production of ROS from SOM in the model is parametrized based on experimental data for monoterpene SOM.^76,77^ Using electron paramagnetic resonance spin trapping with antioxidant-free aqueous solutions and fresh SOM, Tong et al.^76^ found the molar yield of ^•^OH (0.15–1.5%) to be highest for biogenic SOM (i.e., β-pinene, followed by α-pinene, isoprene, and limonene) and negligible for anthropogenic (i.e., naphthalene) SOM.

The model simulations were performed using the NAPS aerosol speciation data as input parameters, specifically the concentrations of water-soluble Fe and Cu, SOM, O_3_, NO_2_, and PM_2.5_.^37,38^ To calculate the SOM concentrations, the contribution of secondary organic carbon (SOC) to the total organic carbon was first calculated for each sample using the OC/EC minimum ratio method, i.e., SOC = OC – (OC/EC)_min_EC.^78,79^ SOC was then converted to SOM using the conversion factor of 1.6 for urban aerosol.^80^ Particle deposition (dose) into the ELF (*D*_PM2.5_; μg mL^–1^) was simulated using the ambient PM_2.5_ concentrations (*C*_PM2.5_; μg m^–3^) and considering a ventilation rate (VR) of 1.5 m^3^ h^–1^, a breathing/accumulation time (*t*_a_) of 2 h, a particle deposition fraction (*d*_PM2.5_) of 0.45 (unitless), and an ELF volume (*V*_ELF_) of 20 mL according to eq 1:^38^
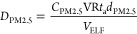
1Further details about the model can be found in the work by Lelieveld et al.,^38^ while the list of reactions included in the model and their rate coefficients are provided in Table S1 in the Supporting Information (SI).

The production of ROS in ELF can be normalized and reported based on (a) the mass of inhaled PM (intrinsic production) and (b) the volume of inhaled air (extrinsic production).^59^ Intrinsic ROS formation reflects the effect of aerosol chemical composition on OP and can be used to assess the effect of the same mass of PM from different aerosol types or sources. The extrinsic ROS formation takes the PM_2.5_ abundance into account; hence, it is site-specific and important in the context of population exposure. In this work, the intrinsic ROS production (pmol min^–1^ μg^–1^) in the lung was calculated from the KM-SUB-ELF output using the reaction time and the PM_2.5_ dose in ELF. The intrinsic values were then multiplied by the ambient PM_2.5_ concentration (μg m^–3^) for each sample to obtain the extrinsic ROS production (pmol min^–1^ m^–3^; also known as oxidative burden). Our previous study found good agreement between KM-SUB-ELF predictions and OP measured using acellular assays.^39^

### 2.5 Estimation of Aerosol pH

The aerosol pH was obtained using the ISORROPIA-Lite (http://isorropia.epfl.ch)^81^ and E-AIM II (http://www.aim.env.uea.ac.uk/aim/aim.php)^82^ aerosol thermodynamic models. The models perform thermodynamic equilibrium calculations for an inorganic aerosol using gaseous and particulate species that affect the water content and ionic composition of the aerosol aqueous phase. Input parameters included ambient temperature (24 h mean), relative humidity (24 h mean), and the concentrations of NH_4_^+^, SO_4_^2–^, NO_3_^–^, NH_3_, and HNO_3_. For pH calculations with the ISORROPIA-Lite model, the concentrations of nonvolatile cations Na^+^, Ca^2+^, Mg^2+^, and K^+^ and organic matter in PM_2.5_ were additionally included to examine their effects on pH. More information about the pH calculations is provided in Section S1 in the SI.

### 2.6 Data Analysis

Statistical analysis and graphical presentation were performed using Origin (OriginLab Corporation, Northampton, U.S.) and Openair R software.^83^ The seasonal differences in aerosol chemical composition, pH, and ROS production were examined using the nonparametric Mann–Whitney test (α = 0.01 or 0.05). The association of OP and aerosol constituents was examined using the nonparametric Spearman rank correlation.

## 3 Results and Discussion

### 3.1 Factors Influencing the Variation in Metal Solubility

#### 3.1.1 Emission Sources

**Figure 1:**
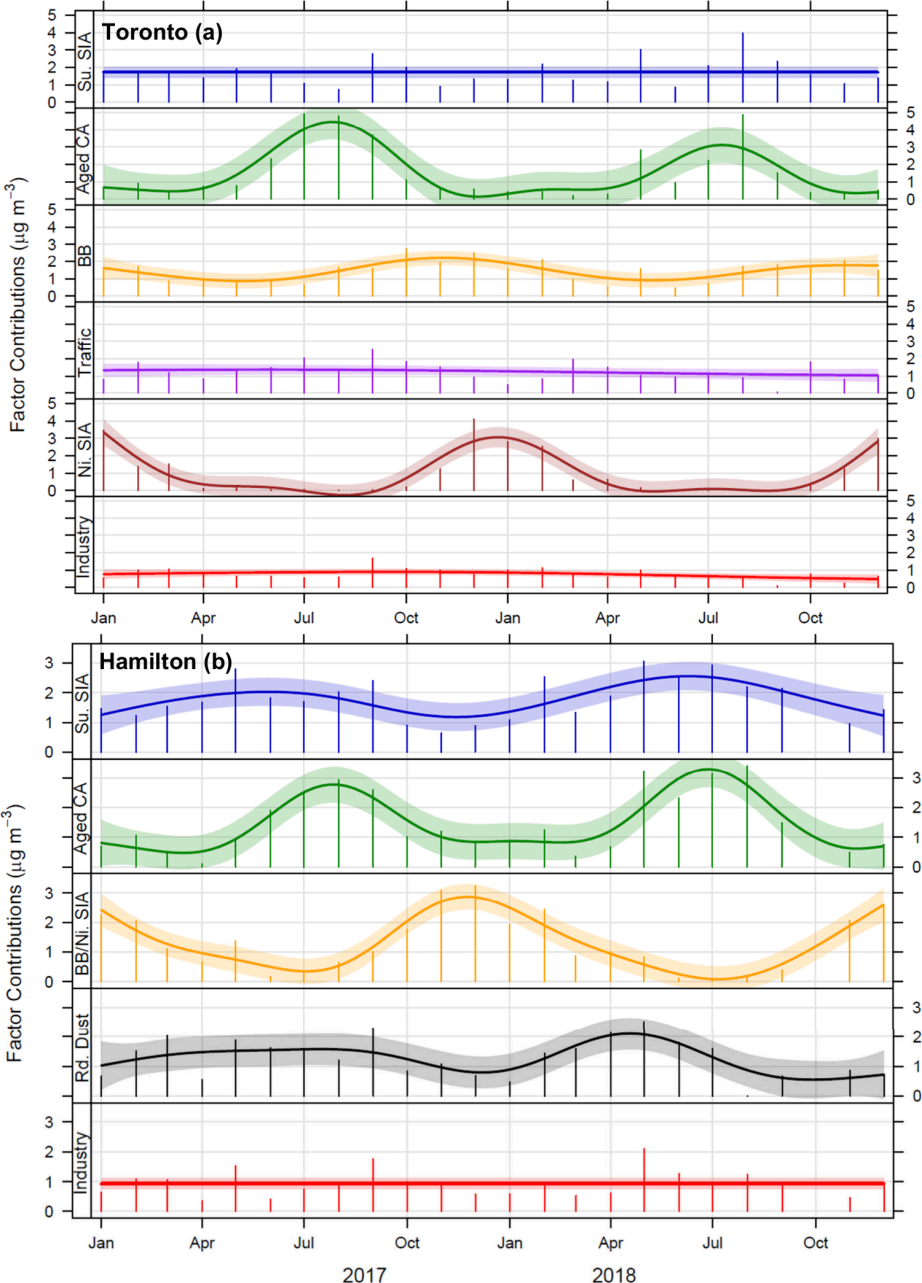
Monthly mean contribution of emission sources to PM_2.5_ mass concentration (μg m^–3^) at Toronto (a) and Hamilton (b) sites obtained using the PMF model. Su. SIA: sulfate secondary inorganic aerosol; Aged CA: aged carbonaceous aerosol; BB: biomass burning; Ni. SIA: nitrate secondary inorganic aerosol; Rd. Dust: road dust (note that this source profile is not shown for the Toronto site because of its negligible contribution to PM_2.5_ mass). The shading shows the estimated 95% confidence interval for the smooth trend.

**Figure 2:**
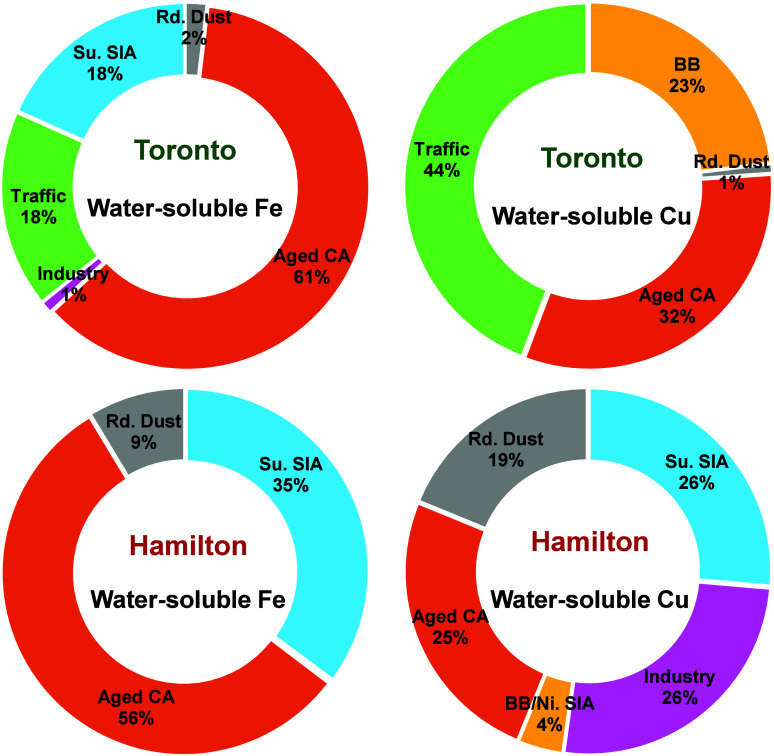
Factor contributions to water-soluble Fe and Cu obtained from PMF analysis of Toronto and Hamilton data (Aged CA: aged carbonaceous aerosol; Su. SIA: sulfate secondary inorganic aerosol; BB: biomass burning; Rd. Dust: road dust; Ni. SIA: nitrate secondary inorganic aerosol). Factors with <1% contribution are not labeled; more information can be found in Tables S2 and S3.

Figure 1 shows the monthly contributions of various sources to PM_2.5_ from the study sites. The source apportionment identified seven factors at the Toronto traffic site, including sulfate secondary inorganic aerosol (SIA; mean contribution: 23%), aged carbonaceous aerosol (19%), biomass burning (19%), traffic (16%), nitrate SIA (13%), industry (10%), and road dust (<1%) (Figures 1a and S1; Table S2). Aged carbonaceous aerosol, on average, made the largest contribution to water-soluble Fe (61%) in Toronto, followed by traffic emissions (18%) and sulfate SIA (18%; Figure 2, Table S2). Aged carbonaceous aerosol was also a major contributor (56%) to oxalate at this site (Table S2). Water-soluble Cu had a rather different source profile in Toronto with the largest contribution coming from traffic emissions (44%), followed by aged carbonaceous aerosol (32%) and biomass burning (23%) (Figure 2, Table S2). In Hamilton, the model identified five factors, including sulfate SIA (mean contribution to PM_2.5_: 26%), aged carbonaceous aerosol (22%), biomass burning/nitrate SIA (19%), road dust (19%), and industry (14%; Figures 1b and S2; Table S3). Similar to Toronto, aged carbonaceous aerosol was the main contributor to water-soluble Fe (56%) in Hamilton, followed by sulfate SIA (35%) and road dust (9%; Figure 2; Table S3). These two sources also had a high association with oxalate (71% and 24%, respectively; Table S3). In contrast, water-soluble Cu was influenced by a more diverse set of sources in Hamilton including industrial emissions (26%), sulfate SIA (26%), aged carbonaceous aerosol (25%), road dust (19%), and biomass burning/nitrate SIA (4%; Figure 2).

Among the main sources that were associated with water-soluble Fe and Cu in Toronto and Hamilton, aged carbonaceous aerosol had the most distinct seasonal trend across the 2017–2018 period, with seasonal means increasing by up to ∼10 times in the summer compared to winter (this increase was twice as large for Toronto compared to Hamilton; Figure 1). The sulfate SIA profile showed a small increase in the summer period only at the Hamilton site, while biomass burning (associated mainly with Cu) showed an increase in the winter period at both sites. It is worth noting that while the aged carbonaceous aerosol profile made similar contributions to water-soluble Fe at both sites (61% vs 56%), the sulfate SIA profile made a relatively higher contribution to Fe in Hamilton (35% vs 18%; Tables S2 and S3). This may indicate that acid dissolution was more important for the Hamilton samples (see section 3.1.2 for a further discussion). The aged carbonaceous aerosol profile is characterized by clustering of OC and EC (from combustion sources) and oxalate (associated with photochemical aging and cloud processing; Figures S1 and S2 ).^46,52,61,64^ The summertime wildfire emissions carried from regions to the north and west of the study areas likely made a small contribution to this source profile, in particular at the Hamilton site (the wood burning tracer levoglucosan contributed 13% to this source profile in Hamilton compared to 2% in Toronto; Tables S2 and S3), as also suggested by a previous study in southern Ontario.^52^

Transboundary air pollution from industrial emissions in the midwestern and eastern U.S. is also expected to contribute to carbonaceous aerosol in southern Ontario, with seasonal enhancement observed during the summer.^52,84^ The enhancement of the aged carbonaceous aerosol profile in the summer is likely influenced by the region’s topography and meteorology, which promote stagnant atmospheric conditions, leading to photochemical aging of air mass fed by combustion sources of local and transboundary origins.^52,61,84^

The notable association of water-soluble Fe with both sulfate SIA and aged carbonaceous aerosol source profiles is related to the formation pathways of sulfate and oxalate through aqueous phase reactions in deliquescent aerosol, fog, and cloud droplets. As a result, the concentrations of the two species typically correlate in ambient PM,^50,64,85^ and this correlation was also relatively high in the present study (*r*_s_ = 0.75–0.86 in Toronto and 0.66–0.84 in Hamilton across various seasons). In fact, oxalate contributed to the sulfate SIA source profile at the Toronto (37%) and Hamilton (24%) sites (Tables S2 and S3). Both sulfate and oxalate could make complexes with Fe in the aerosol aqueous phase and influence the levels of water-soluble Fe.^39,44,48^ Previous studies showed that Fe(III) forms various complexes primarily with oxalate while Fe(II) is present as FeSO_4_ or as free ion in PM_2.5_; these processes and the detailed Fe speciation are influenced by a combination of factors including the aerosol liquid water content, the pH, the levels of organic and inorganic ligands, and the oxidation state of metals.^39,48^ It is worth noting that markedly faster dissolution kinetics were reported previously for PM-bound Fe and Cu in days with fog events and high organic content; this was attributed to aqueous phase processing and metal–ligand complexation with dicarboxylic acids.^48^ Considering the association of Fe and Cu with the aged carbonaceous aerosol profile in the present study and the importance of these metal species for ROS production in the lung, the notable seasonal enhancement of this source profile is expected to increase the PM_2.5_ oxidative burden in the summer period.^33,39^

#### 3.1.2 Proton- and Ligand-Mediated Dissolution of Metals

Figure S3 shows the aerosol pH estimated using ISORROPIA and E-AIM models without the addition of nonvolatile cations and organic matter. The two models provided similar mean pH values at each site for the two-year period, i.e., 2.7 ± 1.0 vs 2.7 ± 0.8 at Toronto and 2.4 ± 0.8 vs 2.5 ± 0.6 at Hamilton. However, ISORROPIA predicted larger variations in pH (interquartile range was 1.5 vs 1.2 at Toronto and 1.2 vs 0.9 at Hamilton; Figure S3), indicating that this model may be more sensitive to changes in input parameters. Hence, we used the ISORROPIA results to explore the parameters that influence metal solubility and OP. To improve the pH estimation, we included the concentrations of particulate nonvolatile cations and organic matter as input parameters (the latter of which can contribute to aerosol water content). The inclusion of these species resulted in a less acidic aerosol (≤0.5 unit increase; Figure S4), less variation in pH, and slightly better agreement between measured and modeled NH_3_ values (which can be used to assess the goodness of model predictions; see Section S1).

**Figure 3:**
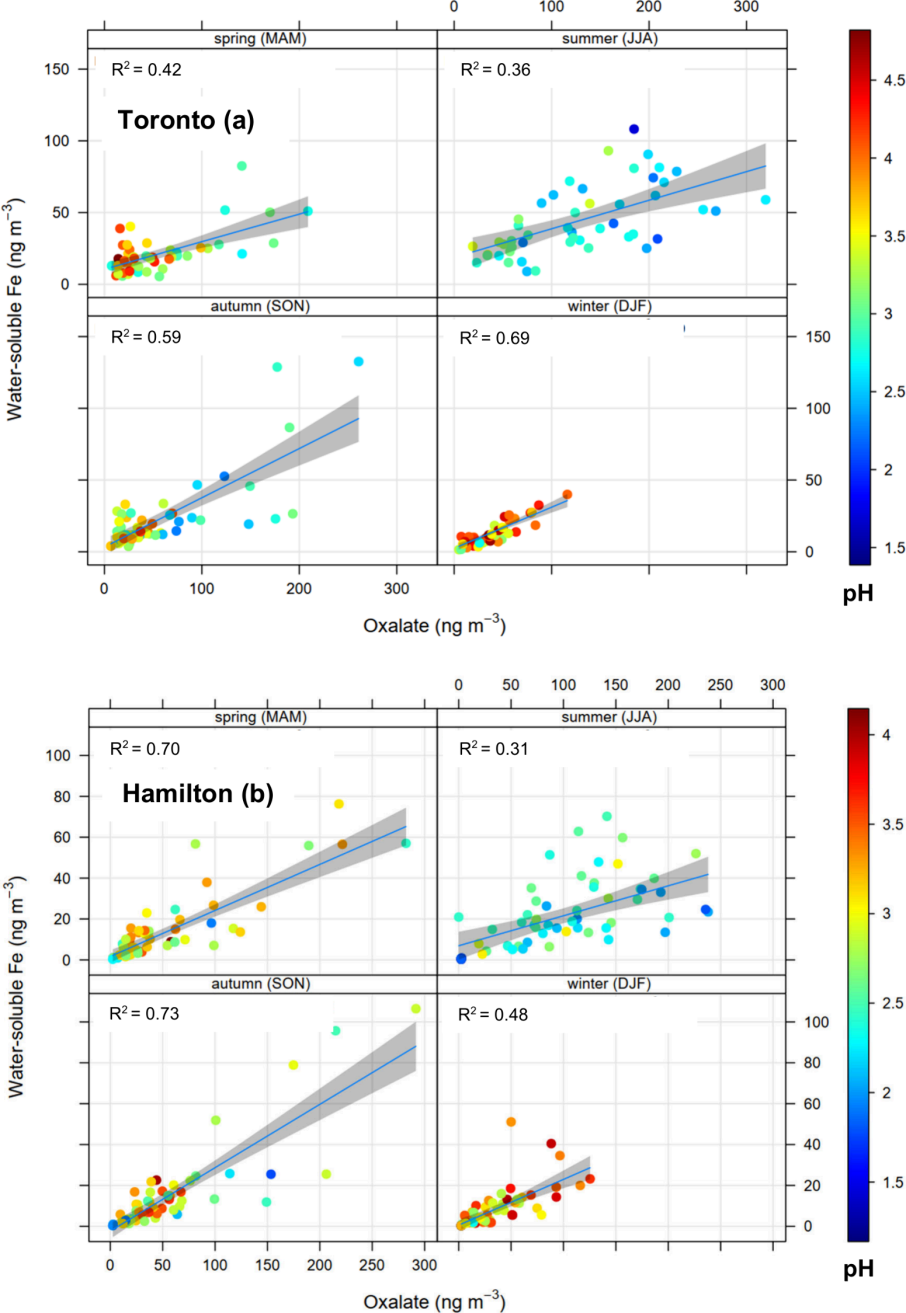
Seasonal variation in oxalate and water-soluble Fe concentrations and aerosol pH at Toronto (a) and Hamilton (b) study sites.

Figure 3a,b shows the seasonal variations in ambient concentration of water-soluble Fe and oxalate, as well as the aerosol pH at the study sites. Oxalate and water-soluble Fe exhibited strong seasonality with the maxima in the summer (Figures 3 and S5a). In Toronto, the mean oxalate concentration was ∼3.5 times higher in summer compared to winter (1.1 × 10^2^ ± 8.8 × 10^1^ vs 32 ± 25 ng m^–3^; Mann–Whitney *p* < 0.01), with similar mean values found in spring and fall (45 ± 48 and 53 ± 56 ng m^–3^, respectively; *p* > 0.05). Similarly, the mean concentration of water-soluble Fe was ∼3.5 times higher in summer compared to winter (39 ± 30 vs 11 ± 9 ng m^–3^; *p* < 0.01), with no significant difference in spring and fall (17 ± 15 and 21 ± 25 ng m^–3^, respectively; *p* > 0.05; Figures 3a and S6a,b). This observation was related mostly to an increase in Fe solubility in summer (median Fe_w_/Fe: 22% vs 10%) (Figures S5a and S6c,d). The aerosol pH also showed a distinct seasonality with the winter mean of 3.7 ± 0.7 compared to 2.8 ± 0.4 in summer (*p* < 0.01) and intermediate values in the spring and fall (3.4 ± 0.7 and 3.1 ± 0.5, respectively; Figure S7).

The summer increase in oxalate levels is due to a higher photochemical production of oxalic acid in the atmosphere,^64,86^ which is reflected by an increase in the levels of O_3_ (Figure S5c). This is further controlled by the magnitude of gas-to-particle conversion of oxalic acid to oxalate, which in turn is influenced by the aerosol pH and ambient temperature,^87^ and stabilization in the aerosol aqueous phase through dissociation and metal–ligand complexation.^39,44,48,50^

In Hamilton, despite a significant difference between summer and winter (*p* < 0.01), a weaker seasonal trend was found for aerosol pH compared to Toronto (∼0.6 vs ∼0.9 unit difference, on average). The summer aerosol in Hamilton was more acidic (pH 2.4 ± 0.4) as was the winter aerosol (3.0 ± 0.4), with similar values in spring and fall (pH 2.6 ± 0.5 and 2.5 ± 0.5, respectively; *p* > 0.05; Figure S7). Moreover, the mean oxalate concentration was ∼3 times higher in summer than in winter (1.1 × 10^2^ ± 7.2 × 10^1^ vs 35 ± 30 ng m^–3^; *p* < 0.01; Figure S5a), close to the values found at the Toronto site. In contrast, the water-soluble Fe concentrations were lower in Hamilton (Figures S5a and S8), and although statistically significant, the summer to winter difference was smaller, i.e., ∼2.5 times (*p* < 0.01) compared to ∼3.5 times difference at the Toronto site. Regardless, similar to the Toronto samples, the Fe soluble fraction (Fe_w_/Fe) in Hamilton increased ∼2-fold in summer compared to the other seasons (median 46% vs 21–31%), with the highest contrast found with winter values (Figure S8). It is interesting to note that the absolute values of Fe_w_/Fe fractions in Hamilton were ∼2 times higher than those of the Toronto samples. This appears to be related to the more acidic aerosol in Hamilton; however, only a weak correlation (*r*_s_ = −0.24) was found between Fe_w_/Fe and the aerosol pH for the individual samples at this site; this correlation was higher for Toronto samples (*r*_s_ = −0.56). Moreover, while oxalate had similar concentration ranges across the two sites (Figures 3 and S5a), the correlation between oxalate and Fe_w_/Fe values was stronger at the Toronto site (*r*_s_ = 0.75 vs 0.56). Hence, the higher Fe_w_/Fe observation at the Hamilton site could be partly related to emissions from high-temperature industrial processes such as metal manufacturing, which is expectedly rich in soluble Fe(II) complexes including FeSO_4_,^88^ compared to other sources of Fe such as mineral dust, which is relatively more important at the Toronto site.^46^ Previous results from the same study sites indicated that ≤45% of Fe(II) would be present as free Fe^2+^ and the rest as FeSO_4_ (with relatively higher Fe^2+^ levels at the Hamilton site).^39^ Furthermore, a higher seasonal variation in aerosol acidity may be responsible for the larger summer to winter difference in water-soluble Fe levels in Toronto (Figure S4).

Seasonal trends were also observed for water-soluble Cu with ∼2 times higher values in summer compared to winter (Toronto: 5.9 ± 4.1 vs 3.0 ± 2.3 ng m^–3^; Hamilton: 3.8 ± 3.6 vs 2.1 ± 2.3 ng m^–3^; *p* < 0.01), with intermediate values in spring and fall (Toronto: 4.1 ± 3.3 and 5.0 ± 3.4 ng m^–3^; Hamilton: 3.1 ± 5.7 and 3.6 ± 3.9 ng m^–3^; *p* > 0.05). We found relatively weak correlations between the concentrations of oxalate and the Cu soluble fraction (i.e., Cu_w_/Cu) at the two sites (*r*_s_ ≤ 0.50; Figures S9 and S10) and no notable association with the aerosol pH (*r*_s_ ≤ −0.29). This is different from the trends seen with Fe and could be explained by the relatively high solubility of Cu irrespective of the aerosol pH or organic ligands. Our results show that the Fe_w_/Fe fraction, on average, could increase by ≤40% from winter to summer, while this increase is only ≤14% for Cu, reaffirming that proton- and ligand-mediated dissolution processes are more important for particulate Fe. Considering the high intrinsic OP and abundance of Fe in ambient air,^75,89^ such dissolution processes could play an important role in regulating the seasonal trends of OP.

### 3.2 Seasonal Variation in ROS Production in the Lung and Implication for Population Health

**Figure 4:**
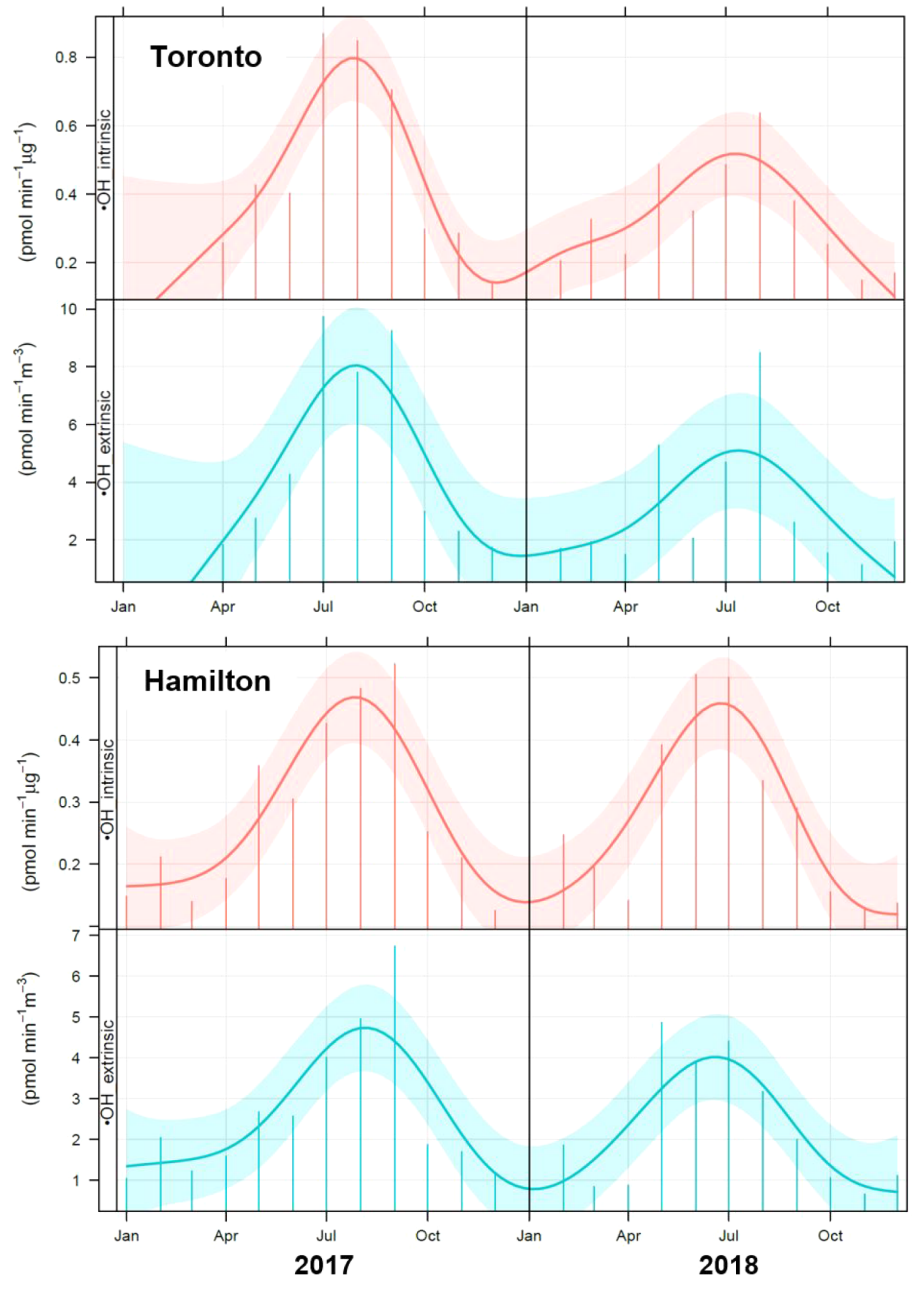
Model-predicted intrinsic (pmol min^–1^ μg^–1^) and extrinsic (pmol min^–1^ m^–3^) ^•^OH production in the lung from the inhalation of ambient air at Toronto and Hamilton sites during the 2017–2018 period. The solid vertical lines represent monthly means (each including 10 data points), while the shading shows the estimated 95% confidence interval for the smoothed trendline.

Figures 4, S11, and S12 show the model-predicted intrinsic and extrinsic ROS production in the lung from the inhalation of ambient air at Toronto and Hamilton sites during the 2017–2018 period. The model-predicted production of O_2_^•–^, H_2_O_2_, and ^•^OH was noticeably lower in the colder months at both sites. The median intrinsic formation rates for O_2_^•–^ and H_2_O_2_ were up to ∼2.5 times lower in winter than in other seasons (O_2_^•–^: 3.2 vs 6.6–8.4 pmol min^–1^ μg^–1^ in Toronto and 2.2 vs 3.4–5.1 pmol min^–1^ μg^–1^ in Hamilton; H_2_O_2_: 1.7 vs 3.0–4.1 pmol min^–1^ μg^–1^ in Toronto and 2.3 vs 2.8–3.3 pmol min^–1^ μg^–1^ in Hamilton; Mann–Whitney *p* < 0.01), while there were no significant differences between the formation rates in summer, spring, and fall (*p* > 0.05). The largest seasonal contrast was found for ^•^OH production in the lung. The median intrinsic ^•^OH formation was up to ∼3.5 times higher in summer compared to the other seasons, particularly winter (0.16 vs 0.27–0.57 pmol min^–1^ μg^–1^ in Toronto and 0.11 vs 0.20–0.38 pmol min^–1^ μg^–1^ in Hamilton; *p* < 0.01). The median winter values were ∼2 times lower than those of spring and fall (*p* < 0.01), while there was no significant difference between the latter two seasons (*p* > 0.05). Similarly, the median extrinsic ROS formation rates in the lung were significantly higher in summer (up to ∼2 times higher in the case of O_2_^•–^ and H_2_O_2_ and up to ∼3.5 times in the case of ^•^OH) compared to other seasons (*p* < 0.01) (Toronto: O_2_^•–^ 73 vs 33–51, H_2_O_2_ 35 vs 18–27, ^•^OH 5.2 vs 1.6–2.2 pmol min^–1^ m^–3^; Hamilton: O_2_^•–^ 42 vs 17–30, H_2_O_2_ 28 vs 14–19, ^•^OH 3.0 vs 0.8–1.3 pmol min^–1^ m^–3^). The largest contrast was seen between winter and summer, with no significant difference between spring and fall (*p* > 0.05). It is interesting to note that the contrasting trends of ^•^OH production in summer and winter closely resemble those of the oxalate and water-soluble Fe concentrations and, in turn, the seasonal changes in the aged carbonaceous aerosol source profile at the two sites (see section 3.1). Moreover, we observed the highest variation in ^•^OH formation in summer and the lowest variation in winter (Figure S11), consistent with the variations in soluble Fe (see the related interquartile ranges in Figures S6b and S8b).

Table 2 shows the contribution of various chemical species to the production of ROS at the two sites. These can be inferred by means of chemical flux analysis with the kinetic model. In this analysis, ROS formation pathways are designated by the responsible chemical components of air pollution. On average, Cu made the largest contribution to the formation of O_2_^•–^ (69–79%) at the two sites, followed by Fe (17–25%) and NO_2_ (2–7%). In contrast, Cu made a small contribution to ^•^OH formation (<0.2%), while the majority of ^•^OH was produced by Fe (56–84%), followed by SOM (16–44%) and negligibly by NO_2_ and O_3_ (<0.2%) in the model.

**Table 2:** Percent Contribution (Mean ± SD) of Inhaled Air Pollutants to Chemical ROS Formation in the Lung

	species	Toronto	Hamilton
O_2_^**•–**^	Cu	73 ± 3	71 ± 15
	Fe	21 ± 4	24 ± 15
	NO_2_	5.9 ± 2.2	5.1 ± 5.3
H_2_O_2_	O_3_	21 ± 6	36 ± 18
	SOM	1.0 ± 0.3	1.2 ± 0.9
	O_2_^•–^a	78 ± 6	63 ± 19
^•^OH^b^	Fe	74 ± 10	62 ± 26
	SOM	26 ± 10	38 ± 26

a Indicates conversion of O_2_^•–^ to H_2_O_2_ through antioxidants and enzymes.

b Cu, NO_2_, and O_3_ made negligible contributions (<0.2%) to ^•^OH formation and are not shown in the table.

While Cu can also form ^•^OH via Fenton-type reactions, the rate constant used in the model for the reaction Fe^2+^ + H_2_O_2_ → Fe^3+^ + ^•^OH + OH^–^ is more than 2 orders of magnitude higher than that for the reaction Cu^+^ + H_2_O_2_ → Cu^2+^ + ^•^OH + OH^–^.^38^ Moreover, water-soluble Fe had notably higher ambient concentrations compared to water-soluble Cu at the two sites during the study period, i.e., 3–7 times (25th–75th percentiles; median: 4) higher at the Toronto site and 2–8 times (median: 4) higher at the Hamilton site. These points explain why Fe was relatively more important for ^•^OH production in the lung in this study. Ozone made a notable contribution to the production of H_2_O_2_ (15–42%) as a product of alkene ozonolysis in the surfactant layer of the ELF. Apart from a small contribution from SOM (<1.5%) and that of O_3_, the model-predicted H_2_O_2_ in the lung originated primarily from conversion of O_2_^•–^ (57–85%) through antioxidants and enzymes (note that endogenous sources of H_2_O_2_ exist in the lung, which may exceed the production capacity of PM_2.5_).^90^

Seasonal changes were also observed in the contribution of chemical species to ROS production. For instance, in Toronto, considering the main contributors to the ^•^OH formation in ELF, water-soluble Fe on average contributed 60% ± 18% in winter and ≤22% more in other seasons, particularly in summer (*p* < 0.01; Figure S13). A similar trend was found for Fe at the Hamilton site, contributing 52% ± 27% to ^•^OH production in winter and up to 20% higher in other seasons, especially in spring and summer (*p* < 0.05; Figure S14). In contrast, despite being more abundant in the warm period (Figure S5a), SOM had its highest relative contribution to ^•^OH production in winter at the Toronto site (40% ± 18%) while making a smaller contribution in summer (18% ± 12%; *p* < 0.01; Figure S13). Our results from Hamilton were somewhat similar, i.e., SOM made a larger contribution to ^•^OH formation in winter (48% ± 27%) and smaller contributions in spring (29% ± 23%), summer, and fall (36% ± 27% and 38% ± 24%; *p* < 0.05). These contrasting results are not surprising, as a large portion of ^•^OH in ELF is produced from Fenton-like reactions involving H_2_O_2_ and the water-soluble Fe^2+^, which is more abundant in summer than in winter (Figure 3a,b), hence reducing the share of SOM to ^•^OH formation.^38,39^ SOM contains high levels of organic hydroperoxides, which could decompose and form substantial amounts of ^•^OH upon encountering water and Fe ions.^76^ While the production of ROS from SOM in KM-SUB-ELF (i.e., the sum of H_2_O_2_ and ^•^OH) is parametrized based on fresh SOM in antioxidant-free aqueous solutions,^37,38,76,77^ SOM-bound organic peroxides have been shown to decompose within several hours,^91,92^ which suggests that aged SOM may generate lower quantities of ROS. Moreover, recent reports showed that SOM may produce other radical species (e.g., O_2_^•–^) depending on SOM type and composition,^93^ and organic radicals,^94^ especially in the presence of antioxidants. These new insights demand revisiting the SOM treatment in KM-SUB-ELF in future studies.

Overall, the ∼2 times higher intrinsic ^•^OH production found at the Toronto traffic site is consistent with our previous results from the two study locations using various acellular OP assays.^39,46^ This is related to higher mass mixing ratios of pollutants, in particular Fe and Cu in PM_2.5_, at the Toronto site, which is due in part to its proximity (∼10 m) to a major highway with a continuous flow of light- and heavy-duty traffic (annual average daily traffic of ∼400 000 vehicles).^55^ Regardless of the intersite differences in intrinsic OP, our findings indicate three conditions that regulate the seasonal changes in the oxidative burden of ambient air in the traffic and industrial sites of Toronto and Hamilton: (a) chemically aged and highly acidic aerosol in summer combined with a high abundance of organic ligands and water-soluble metals results in elevated levels of ROS, particularly ^•^OH, in the lung. (b) Opposite conditions in winter lead to a relatively low oxidative burden, and (c) intermediate conditions in spring and fall result in a moderate oxidative burden. These conditions will have important implications for public health in urban areas, particularly for those residing in the vicinity of traffic and industrial emission sources. In warm periods, populations can be negatively affected by the elevated oxidative burden of ambient air, independent of the emission sources considered in this work. Moreover, synergistic effects from the interaction of Fe and Cu with particulate organic matter and increased levels of reactive species such as peroxides and quinones could further increase OP.^95,96^

A higher oxidative burden in summer, compared to the annual mean, was also reported in a previous study conducted across urban sites in Toronto, where constant fractional solubility was assumed for Fe and Cu as the only pollutants for estimating ROS formation.^33^ The study attributed the contrasting seasonal pattern to a higher ambient concentration of metals in summer due to reduced precipitation. Our results show that the increase in ROS production is also affected by the enhanced solubility of metals and, in particular, Fe in the summer months due to aerosol photochemical aging and aqueous phase chemistry. We presume that Fe dissolution is suppressed in winter following less acidic aerosol and low levels of organic ligands.^50^

One must note that differences can be expected between model predictions and acellular OP assays since the conditions involved in these approaches are not identical; the underlying chemistry of the assays and the diversity of analytical protocols can affect the observed OP trends (see the work by Shahpoury et al.^39^ for further discussions). Site-specific emission sources and local atmospheric conditions can further modify the seasonal trends of OP. For instance, contrasting seasonal trends were reported with the dithiothreitol assay,^23,97−102^ including higher extrinsic OP in the winter, which can be attributed to the relatively high reactivity of dithiothreitol to organic species,^21,23,103,104^ elevated emissions of organic aerosols from residential heating (biomass burning), and low boundary layer height.

Among the species considered in the kinetic model in the present work, Fe, Cu, SOM, and O_3_ made the largest contribution to various forms of ROS; however, water-soluble Fe is the most important pollutant for the production of highly reactive ^•^OH in the model, and indeed, it was previously linked to acute cardiovascular effects from the inhalation of urban PM_2.5_.^105^ Fe may require particular attention in future emission control programs.^39,59^ The results from this study show that the oxidative burden from the inhalation of Fe alone can increase ∼1–2 orders of magnitude from winter to summer (i.e., a 0.15 unit increase in OP for a 1 ng m^–3^ increase in water-soluble Fe). Such an increase in OP is concerning, considering that summer months coincide with long periods of wildfire emissions, which have been increasing in Canada and around the globe.^106^ Wildfire smoke contains large amounts of gaseous and particulate pollutants including metals and organic species, and they could further aggravate the negative health effects of air pollution on populations.^107,108^ Future research should look more closely at these concurring processes and their effect on air quality.

## Supporting Information Available

List of reactions included in KM-SUB-ELF, description of pH estimation methods and comparison of E-AIM and ISORROPIA-Lite models, further information about the PMF source apportionment, seasonal changes in the concentrations of aerosol chemical species, Fe and Cu soluble concentrations and solubility, ambient temperature and aerosol acidity, model-predicted ROS production, and contribution of air pollutants to ROS production (PDF)
