# Supplementary material for: Seasonal Changes in the Oxidative Potential of Urban Air Pollutants: The Influence of Emission Sources and Proton- and Ligand-Mediated Dissolution of Transition Metals
Source: ACS EST Air. 2024 Aug 29;1(10):1262–75. doi: 10.1021/acsestair.4c00093 (PMC11474821; doi:10.1021/acsestair.4c00093)
Supplement: Supplementary file 1 — ea4c00093_si_001.pdf [file ea4c00093_si_001.pdf]

Supporting information for

Seasonal changes in the oxidative potential of urban air pollutants – the influence of emission sources, and proton- and ligand-mediated dissolution of transition metals

Pourya Shahpoury,[1]* Steven Lelieveld,[2] Deepchandra Srivastava,[3] Andrea Baccarini,[4] Jacob Mastin,[5] Thomas Berkemeier,[2] Valbona Celo,[6] Ewa Dabek-Zlotorzynska,[6] Tom Harner,[5]* Gerhard Lammel,[2] Athanasios Nenes[4,7]

[1] Environmental and Life Sciences, Trent University, Peterborough, Canada

[2] Multiphase Chemistry Department, Max Planck Institute for Chemistry, Mainz, Germany

[3] Division of Environmental Health and Risk Management, School of Geography, Earth & Environmental Sciences, University of Birmingham, Edgbaston, Birmingham, UK

[4] Laboratory of Atmospheric Processes and their Impacts, School of Architecture, Civil and Environmental Engineering, École Polytechnique Fédérale de Lausanne, Switzerland

[5] Air Quality Processes Research Section, Environment and Climate Change Canada, Toronto, Canada

[6]Analysis and Air Quality Section, Environment and Climate Change Canada, Ottawa, Canada

[7] Institute of Chemical Engineering Sciences, Foundation for Research and Technology Hellas, Patras, Greece

Correspondence: Pourya Shahpoury (pshahpoury@trentu.ca); Tom Harner (tom.harner@ec.gc.ca)

Contents

Table S1. Chemical reactions and rate coefficients used in KM-SUB-ELF 3

Section S1. Estimation of aerosol pH 6

Figure S1: Factor contributions from the PMF analysis of Toronto data 11

Table S2. Factor profiles (% of species) from PMF analysis of Toronto data....12

Figure S2: Factor contributions from the PMF analysis of Hamilton data 14

Table S3. Factor profiles (% of species) from PMF analysis of Hamilton data 15

Figure S3. Comparison of aerosol pH obtained from ISORROPIA and E-AIM models 16

Figure S4. aerosol pH obtained using ISORROPIA model 16

Figure S5a. Concentrations of Cu, Fe, and their water-soluble forms Cu_w and Fe_w, and oxalate 17

Figure S5b. Concentrations of sulfate, nitrate, ammonium, organic and elemental carbon 18

Figure S5c. Concentrations of $PM_{2.5}$, levoglucosan, nitrogen dioxide, and ozone 19

Figure S6. Seasonal variation in oxalate and water-soluble Fe concentrations, Fe solubility, oxalate mass fraction, and aerosol pH at Toronto site 20

Figure S7. Seasonal changes in ambient temperature and aerosol pH....21

Figure S8. Seasonal variation in oxalate and water-soluble Fe concentrations, Fe solubility, oxalate mass fraction, and aerosol pH at Hamilton site....22

Figure S9. Seasonal variation in oxalate and water-soluble Cu concentrations, Cu solubility, oxalate mass fraction, and aerosol pH at Toronto site........23

Figure S10. Seasonal variation in oxalate and water-soluble Cu concentrations, Cu solubility, oxalate mass fraction, and aerosol pH at Hamilton site........24

Figure S11. ROS production in the lung from the inhalation of ambient air at Toronto site........25

Figure S12. ROS production in the lung from the inhalation of ambient air at Hamilton site........26

Figure S13. Contribution of various chemical species to ROS formation at Toronto site........27

Figure S14. Contribution of various chemical species to ROS formation at Hamilton site........29

References........31

Table S1. Chemical reactions and rate coefficients used in KM-SUB-ELF

#	Reaction	Rate coefficient (cm^{-3} s^{-1} or s^{-1})
	Gas-phase reactions	
1	$NO\bullet + O_3 \rightarrow NO_2\bullet + O_2$	2.05×10^{-14}
2	$NO_2\bullet + O_3 \rightarrow NO_3\bullet + O_2$	4.85×10^{-17}
3	$NO\bullet + NO\bullet + O_2 \rightarrow NO_2\bullet + NO_2\bullet$	8.93×10^{-20}
4	$NO\bullet + NO_3\bullet \rightarrow NO_2\bullet + NO_2\bullet$	2.57×10^{-11}
5	$NO_2\bullet + NO_3\bullet \rightarrow NO\bullet + NO_2\bullet + O_2$	7.73×10^{-16}
6	$NO_2\bullet + NO_3\bullet \rightarrow N_2O_5$	1.21×10^{-12}
7	$\bullet OH + O_3 \rightarrow HO_2\bullet + O_2$	8.20×10^{-14}
8	$\bullet OH + H_2O_2 \rightarrow HO_2\bullet + H_2O$	1.73×10^{-12}
9	$HO_2\bullet + O_3 \rightarrow \bullet OH + O_2 + O_2$	8.24×10^{-16}
10	$\bullet OH + HO_2\bullet \rightarrow H_2O + O_2$	1.08×10^{-10}
11	$HO_2\bullet + HO_2\bullet \rightarrow H_2O_2 + O_2$	5.09×10^{-12}
12	$HO_2\bullet + HO_2\bullet \rightarrow H_2O_2 + O_2$	3.50×10^{-12}
13	$\bullet OH + NO\bullet \rightarrow HONO$	8.91×10^{-12}
14	$\bullet OH + NO_2\bullet \rightarrow HNO_3$	8.91×10^{-12}
15	$\bullet OH + NO_3\bullet \rightarrow HO_2\bullet + NO_2\bullet$	2.00×10^{-11}
16	$HO_2\bullet + NO\bullet \rightarrow \bullet OH + NO_2\bullet$	8.24×10^{-12}
17	$HO_2\bullet + NO_2\bullet \rightarrow HO_2NO_2$	6.87×10^{-13}
18	$HO_2NO_2 \rightarrow HO_2\bullet + NO_2\bullet$	2.49×10^{-1}
19	$\bullet OH + HO_2NO_2 \rightarrow NO_2\bullet + H_2O + O_2$	2.96×10^{-12}
20	$HO_2\bullet + NO_3\bullet \rightarrow \bullet OH + NO_2\bullet + O_2$	4.00×10^{-12}
21	$\bullet OH + HONO \rightarrow NO_2\bullet + H_2O$	5.78×10^{-12}
22	$\bullet OH + HNO_3 \rightarrow NO_3\bullet + H_2O$	1.37×10^{-13}
23	$N_2O_5 \rightarrow NO_2\bullet + NO_3\bullet$	1.83×10^{-1}
	Surfactant reactions	
24	$SPB + \bullet OH \rightarrow SPB\text{-}ox$	1.70×10^{-11}
25	$POG + \bullet OH \rightarrow POG\text{-}ox$	1.70×10^{-11}
26	$SPB + O_3 \rightarrow SPB\text{-}ox$	1.00×10^{-14}
27	$POG + O_3 \rightarrow POG\text{-}ox + 0.17\ H_2O_2$	1.66×10^{-16}
28	$aToc + OH \rightarrow aToc\text{-}ox$	4.50×10^{-13}
29	$aToc + O_3 \rightarrow aToc\text{-}ox$	1.20×10^{-18}
	ELF reactions	
30	$O_2\bullet^- + HO_2 + H_2O \rightarrow H_2O_2 + OH^- + O_2$	1.70×10^{-13}
31	$HO_2 + HO_2 \rightarrow H_2O_2 + O_2$	1.40×10^{-15}
32	$O_2\bullet^- + O_2\bullet^- + 2H^+ \rightarrow H_2O_2 + O_2$	3.82×10^{-16}

Table S1 continued – *ELF reactions*

33	$H_2O_2 + {\bullet}OH \rightarrow HO_2 + H_2O$	5.50×10^{-14}
34	${\bullet}OH + {\bullet}OH \rightarrow H_2O_2$	8.60×10^{-12}
35	${\bullet}OH + O_2{\bullet}^- \rightarrow O_2 + OH^-$	1.30×10^{-11}
36	${\bullet}OH + HO_2 \rightarrow H_2O + O_2$	1.20×10^{-11}
37	$H_2O_2 + HO_2 \rightarrow {\bullet}OH + O_2 + H_2O$	4.98×10^{-21}
38	$Fe^{2+} + O_2^- + 2H^+ \rightarrow Fe^{3+} + H_2O_2$	3.10×10^{-14}
39	$Fe^{2+} + HO_2 + H^+ \rightarrow Fe^{3+} + H_2O_2$	1.99×10^{-15}
40	$Fe^{2+} + H_2O_2 \rightarrow Fe^{3+} + {\bullet}OH + OH^-$	4.30×10^{-18}
41	$Fe^{2+} + {\bullet}OH \rightarrow Fe^{3+} + OH^-$	5.30×10^{-13}
42	$Fe^{2+} + H_2O_2 \rightarrow Fe^{4+} + H_2O$	9.50×10^{-18}
43	$Fe^{3+} + H_2O_2 \rightarrow Fe^{2+} + HO_2 + H^+$	3.32×10^{-24}
44	$Fe^{3+} + HO_2 \rightarrow Fe^{2+} + O_2 + H^+$	3.30×10^{-18}
45	$Fe^{4+} + Fe^{2+} \rightarrow Fe^{3+} + Fe^{3+}$	6.60×10^{-18}
46	$Fe^{3+} + AscH \rightarrow Fe^{2+} + Asc{\bullet}$	1.10×10^{-19}
47	$Fe^{4+} + AscH \rightarrow Fe^{3+} + Asc{\bullet}$	7.60×10^{-19}
48	$Fe^{2+} + O_2 \rightarrow O_2{\bullet}^- + Fe^{3+}$	5.20×10^{-21}
49	$Cu^+ + HO_2 + H^+ \rightarrow Cu^{2+} + H_2O_2$	2.30×10^{-12}
50	$Cu^+ + O_2{\bullet}^- + H_2O \rightarrow Cu^{2+} + H_2O_2 + OH^-$	5.80×10^{-15}
51	$Cu^{2+} + HO_2 \rightarrow Cu^+ + O_2 + H^+$	1.60×10^{-11}
52	$Cu^{2+} + O_2{\bullet}^- \rightarrow Cu^+ + O_2$	8.30×10^{-12}
53	$Cu^{2+} + AscH \rightarrow Cu^+ + Asc{\bullet}$	1.40×10^{-18}
54	$Cu^+ + O_2 \rightarrow Cu^{2+} + O_2{\bullet}^-$	6.90×10^{-20}
55	$Cu^+ + H_2O_2 \rightarrow Cu^{2+} + {\bullet}OH + OH^-$	2.40×10^{-20}
56	$Cu^+ + H_2O_2 \rightarrow Cu^{3+} + OH^- + OH^-$	5.00×10^{-19}
57	$Cu^+ + Cu^{3+} \rightarrow Cu^{2+} + Cu^{2+}$	5.80×10^{-12}
58	$Cu^{2+} + H_2O_2 \rightarrow Cu^+ + O_2{\bullet}^- + H^+$	3.80×10^{-24}
59	$PQN + AscH \rightarrow PQN{\bullet} + Asc{\bullet}$	1.20×10^{-20}
60	$PQN{\bullet} + O_2 \rightarrow PQN + O_2{\bullet}^-$	4.60×10^{-13}
61	$PQN{\bullet} + O_2{\bullet}^- + 2H^+ \rightarrow PQN + H_2O_2$	3.30×10^{-12}
62	$NQN12 + AscH \rightarrow NQN12{\bullet} + Asc{\bullet}$	1.50×10^{-19}
63	$NQN12{\bullet} + O_2 \rightarrow NQN12 + O_2{\bullet}^-$	4.60×10^{-13}
64	$NQN12{\bullet} + O_2{\bullet}^- + 2H^+ \rightarrow NQN12 + H_2O_2$	3.30×10^{-12}
65	$NQN14 + AscH \rightarrow NQN14{\bullet} + Asc{\bullet}$	6.30×10^{-21}
66	$NQN14{\bullet} + O_2 \rightarrow NQN14 + O_2{\bullet}^-$	4.60×10^{-13}
67	$NQN14{\bullet} + O_2{\bullet}^- + 2H^+ \rightarrow NQN14 + H_2O_2$	3.30×10^{-12}
68	$UA + O_3 \rightarrow Products$	9.60×10^{-17}
69	$UA + {\bullet}OH \rightarrow Products + OH^-$	1.20×10^{-11}

Table S1 continued – *ELF reactions*

70	GSH + •OH → Products + OH^-	1.50×10^{-11}
71	GSSG + •OH → Products + OH^-	1.50×10^{-11}
72	Asc• + Asc• + H^+ → AscH + DHA	5.00×10^{-16}
73	AscH + O_2•$^-$ + H^+ → Asc• + H_2O_2	5.10×10^{-17}
74	AscH + HO_2 → Asc• + H_2O_2	2.65×10^{-17}
75	AscH + •OH → Products + OH^-	1.80×10^{-11}
76	AscH + •O_3 → Products	9.10×10^{-17}
77	1.25 GS^- + 0.5 O_3 → Products	9.60×10^{-20}
78	1.25 GSH + 0.5 O_3 → Products	9.60×10^{-20}
79	GSOO + GSOO → 0.56 O_2•$^-$ + Products	6.79×10^{-13}
80	O_2•$^-$ + GSH → GSO• + OH^-	3.32×10^{-19}
81	NO_2• + GS• → $GSNO_2$	4.98×10^{-12}
82	GSOO• + NO_2• → $GSOONO_2$	2.49×10^{-12}
83	$GSOONO_2$ → GSOO• + NO_2•	7.5×10^{-1}
84	NO_2• + GS^- → NO_2^- + GS•	4.00×10^{-13}
85	NO_2• + GSH → NO_2^- + GS• + H^+	1.66×10^{-14}
86	GSOO• + GSH → GSO• + GSOH	3.32×10^{-15}
87	GSO + NO_2 → GSOONO	7.47×10^{-12}
88	GSOONO → Products	7.00×10^{2}
89	GS• + GS^- → GSSG•$^-$	1.59×10^{-14}
90	GSSG•$^-$ → GS• + GS^-	1.60×10^{5}
91	GSSG•$^-$ + O_2 → GSSG + O_2•$^-$	8.30×10^{-12}
92	GS• + GS• → GSSG	8.30×10^{-12}
93	GSOH + GSH → GSSG + H_2O	1.20×10^{-18}
94	GSO• + GSO• → Products	9.96×10^{-14}
95	GS^- + H_2O_2 → GSOH + OH^-	1.60×10^{-21}
96	GS• + AscH → GSH + Asc•	1.00×10^{-12}
97	UA + NO_2• → UA• + NO_2^-	3.00×10^{-14}
98	AscH + NO_2• → Asc• + NO_2^-	5.80×10^{-14}
99	UA• + AscH → UA + Asc•	1.70×10^{-15}
100	GS• + UA → GSH + UA•	5.00×10^{-14}
101	O_2•$^-$ + NO_2• → O_2NOOm	7.50×10^{-12}
102	O_2NOO^- → NO_2^- + O_2	7.00×10^{-1}
103	O_2NOO^- → O_2•$^-$ + NO_2•	1.10×10^{0}
104	NO_2• + NO_2• → N_2O_4	7.50×10^{-13}
105	N_2O_4 → NO_2• + NO_2•	6.90×10^{3}
106	N_2O_4 + H_2O → NO_2^- + NO_3^- + $2H^+$	1.00×10^{3}

Table S1 continued – *ELF reactions*

107	$O_2^- + O_3 + H_2O \rightarrow \bullet OH + 2O_2 + OH^-$	2.50×10^{-12}
108	$HO_2 + O_3 \rightarrow \bullet OH + 2O_2$	1.66×10^{-17}
109	$NO_2^- + \bullet OH \rightarrow NO_2\bullet + OH^-$	8.80×10^{-12}
110	$\bullet OH + NO_2\bullet \rightarrow NO_3^- + H^+$	7.50×10^{-12}
111	$\bullet OH + NO_2\bullet \rightarrow$ ONOOH	7.50×10^{-12}
112	ONOOH $\rightarrow NO_2\bullet + \bullet OH$	3.00×10^{-1}
113	ONOOH $\rightarrow NO_3^- + H^+$	7.00×10^{-1}
114	$ONOO^-$ + GSH $\rightarrow NO_2^-$ + GSOH	1.10×10^{-18}
115	GSO• + $NO_2\bullet \rightarrow$ GSOONO	7.50×10^{-12}
116	GSOONO + $H_2O \rightarrow$ Products	7.00×10^{2}
117	ONOOH + AscH $\rightarrow Im_1$	1.66×10^{-15}
118	$Im_1 \rightarrow$ ONOOH + AscH	5.00×10^{2}
119	$Im_1 \rightarrow Im_2$	4.00×10^{1}
120	$Im_2 \rightarrow Im_1$	5.00×10^{0}
121	Im_2 + AscH $\rightarrow$ Asc + DHA + $NO_2^- + H_2O$	1.66×10^{-19}
122	$Im_2 \rightarrow$ Asc + $NO_3^- + H^+$	8.50×10^{-1}
123	ONOOH + UA $\rightarrow UA^{rad} + NO_2$ + Products	2.60×10^{-19}
124	$O_2\bullet^-$ + SOD + $H^+ \rightarrow 0.5\ H_2O_2$ + SOD	2.65×10^{-12}
125	H_2O_2 + catalase $\rightarrow H_2O + 0.5\ O_2$ + catalase	3.20×10^{-14}
126	•OH + organic matter $\rightarrow$ oxidized organic matter	1.66×10^{-12}

Section S1. Estimation of aerosol pH

As a direct measurement of aerosol pH is not possible,[1] the most reliable estimates can be obtained through thermodynamic analysis of ambient observations.[1–3] This approach involves the application of a thermodynamic model to observations of the major inorganic species in the gas and particulate phase that affect the water content and ionic composition in the aqueous phase present in the aerosol. In this study, the aerosol pH was calculated using both ISORROPIA-Lite and E-AIM II models.[4,5] The models perform thermodynamic equilibrium calculation for an inorganic aerosol system. For instance, ISORROPIA-Lite determines a set of sub-system equilibrium equations and solves the equations for equilibrium state using the chemical potential method.[6,7] The modelled aerosol system consists of potential components in the gas-phase, i.e. NH_3, HNO_3, HCl, H_2O, in the liquid-phase, i.e. NH_4, Na^+, H^+, Cl^-, NO_3^-, SO_4^{2-}, HNO_3, NH_3, HCl, HSO_4^-, OH^-, H_2O, Ca^{2+}, K^+, Mg^{2+}, and in the solid phase, i.e. $(NH_4)_2SO_4$, NH_4HSO_4, $(NH_4)_3H(SO_4)_2$, NH_4NO_3, NH_4Cl, NaCl, $NaNO_3$, $NaHSO_4$, Na_2SO_4, $CaSO_4$, $Ca(NO_3)_2$, $CaCl_2$, K_2SO_4, $KHSO_4$, KNO_3, KCl, $MgSO_4$, $Mg(NO_3)_2$, and $MgCl_2$. ISORROPIA-Lite also includes the effect of organics on the aerosol liquid water content which is parametrized in terms of organics mass and hygroscopicity. The model calculates the pH by estimating the equilibrium concentration of H^+ and liquid water content (LWC) in the aerosol, and the pH is calculated using the "pH_F" definition of Pye et al. (2020), Eq. S1,[1]

$$pH = -\log_{10} \gamma_{H^+} H^+_{aq} \cong -\log_{10} \frac{1000\gamma_{H^+} H^+_{air}}{W} \qquad \text{Eq. S1}$$

where γ_{H+} is the activity coefficient of the hydronium ion, H^+ (assumed unity), H_{aq}^+ is its concentration (mol L^{-1}) in the aerosol aqueous phase, H_{air}^+ (μg m^{-3}) is the concentration of H^+ per volume of air, and W (μg m^{-3}) is particle water concentrations associated with both the organic and inorganic aerosol species. With ISORROPIA-Lite, input parameters consisted of aerosol precursors NH_4^+, NH_3, SO_4^{2-}, NO_3^-, HNO_3, Na, Ca, Mg, K, mass, density and hygroscopicity of organics (treated as one single phase), as well as ambient temperature and relative humidity. For this study, the hygroscopicity of organics was assumed to be equal to 0.15 and the density to 1.4 g cm^{-3}. The inclusion of the crustal material (Na, Ca, Mg, and K) in the model improves the prediction of ammonium and nitrate partitioning in the aerosol and the estimation of pH, particularly when crustal dust constitutes a considerable fraction of $PM_{2.5}$.

With the E-AIM II model, pH was calculated using Eq. S2,[8] with NH_4^+, NH_3, SO_4^{2-}, NO_3, and, as well as ambient temperature and relative humidity as input parameters.

$$pH = -\,log_{10}(a_H^+) = \; -\,log_{10}(f_H^+ \times x_H^+ \times 55.509) \qquad \text{Eq. S2}$$

a_H^+ is the activity of H^+ in aerosol LWC, f_H^+ is the H^+ mole-fraction-based activity coefficient, x_H^+ is the H^+ mole fraction, and 55.509 is the conversion factor from a mole-based activity coefficient to a molality-based coefficient.[9] The model was set to avoid formation of solid phases, i.e. the condition of metastable,

supersaturated, and aqueous aerosol. Without the inclusion of non-volatile cations and organic matter in the calculations, the results of E-AIM and ISORROPIA models were comparable, with mean values of 2.67±0.99 vs. 2.68±0.83 at Toronto and 2.43±0.77 vs. 2.48±0.62 at Hamilton site. ISORROPIA captured a larger variation in data compared to E-AIM (shown by the interquartile ranges at the two sites, i.e. 1.52 vs. 1.20 and 1.18 vs. 0.85 at Toronto and Hamilton, respectively). With ISORROPIA, the inclusion of non-volatile cations increased the mean pH values by 0.54 and 0.27 units at Toronto (pH 3.21±1.23) and Hamilton (pH 2.70±0.81) sites, respectively, whereas the addition of organic matter resulted in only 0.02 and 0.06 units increase in pH at the two sites, respectively. Compared to the base case, including non-volatile cations and organic matter resulted in smaller seasonal variation in pH (the interquartile range decreased from 1.18-1.52 to 0.76-1.12 at the two sites; Figure S3-S4). The ratio of model-predicted to measured NH_3 is typically used to assess the goodness of pH predictions. Our results from Toronto showed that, with non-volatile cations and organic matter included, this ratio increased on average from 0.93 to 0.97, compared to the case without non-volatile cations and organic matter, whereas this change was negligible for the Hamilton site (~0.86) due to lower concentrations of non-volatile cations at this site.

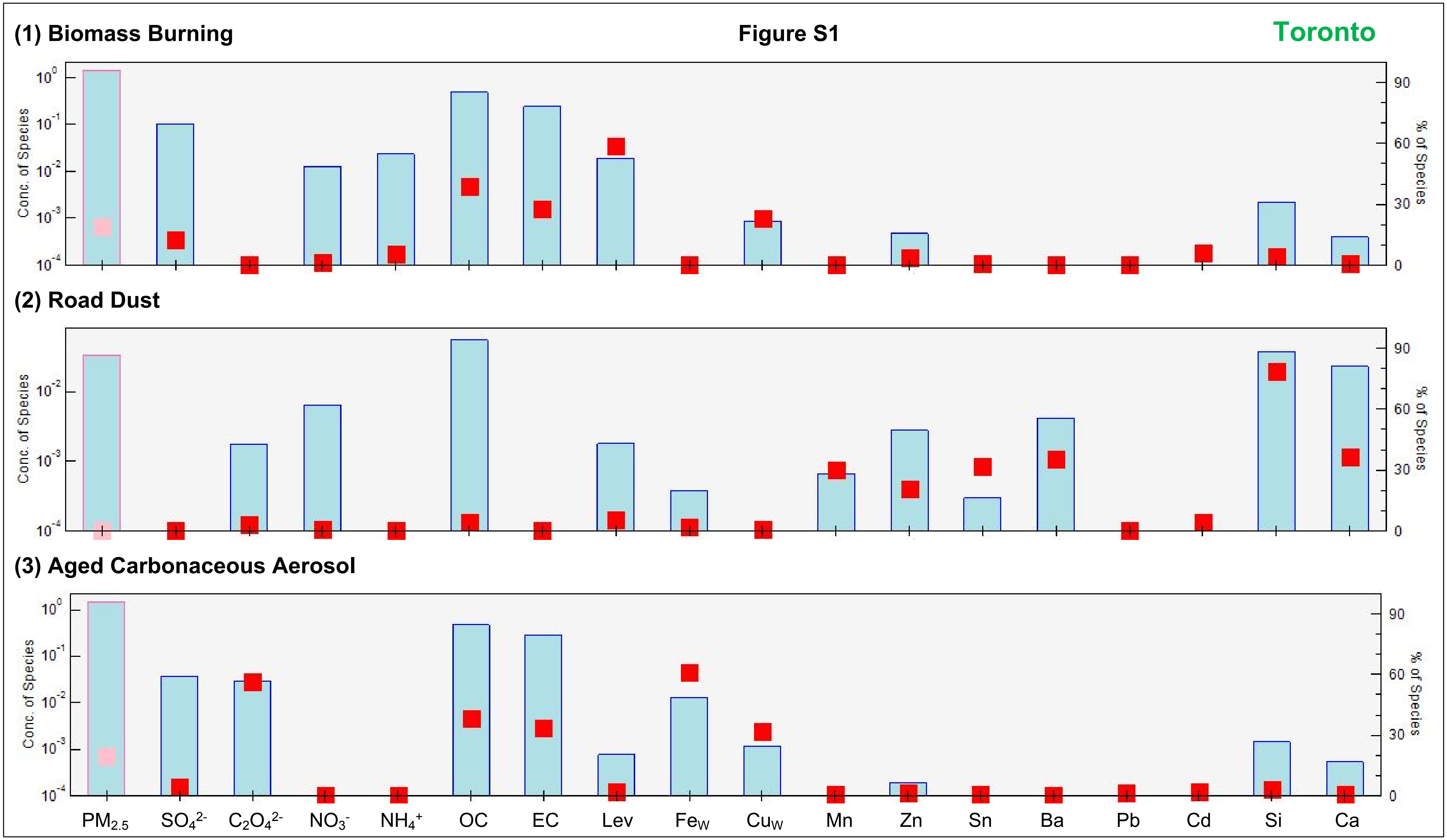
(1) Biomass Burning
Figure S1
Toronto
Conc. of Species
% of Species
(2) Road Dust
(3) Aged Carbonaceous Aerosol
PM2.5
SO4 2-
C2O4 2-
NO3 -
NH4 +
OC
EC
Lev
FeW
CuW
Mn
Zn
Sn
Ba
Pb
Cd
Si
Ca

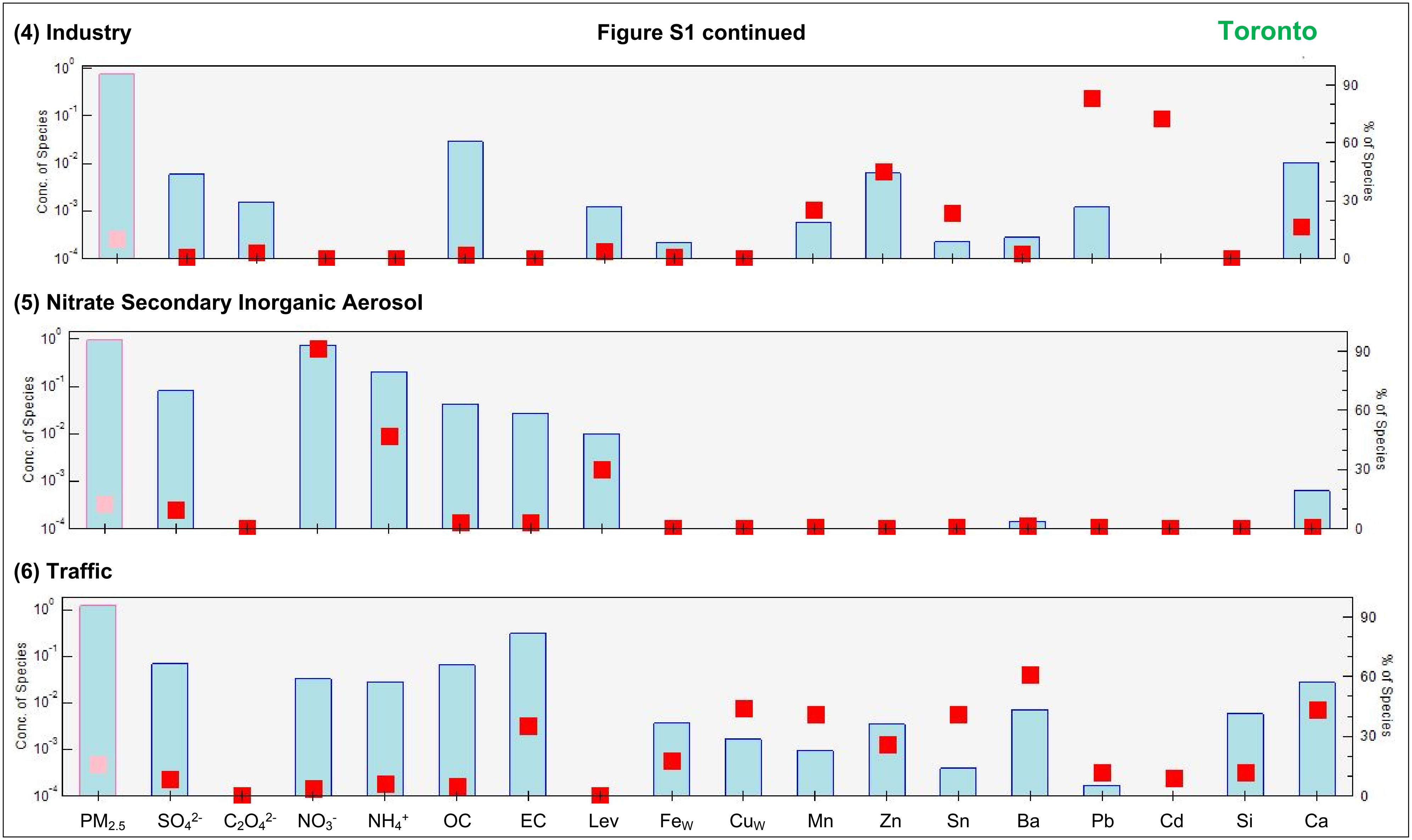
(4) Industry
Figure S1 continued
Toronto
(5) Nitrate Secondary Inorganic Aerosol
(6) Traffic
Conc. of Species
% of Species
PM2.5
SO4 2-
C2O4 2-
NO3 -
NH4 +
OC
EC
Lev
FeW
CuW
Mn
Zn
Sn
Ba
Pb
Cd
Si
Ca

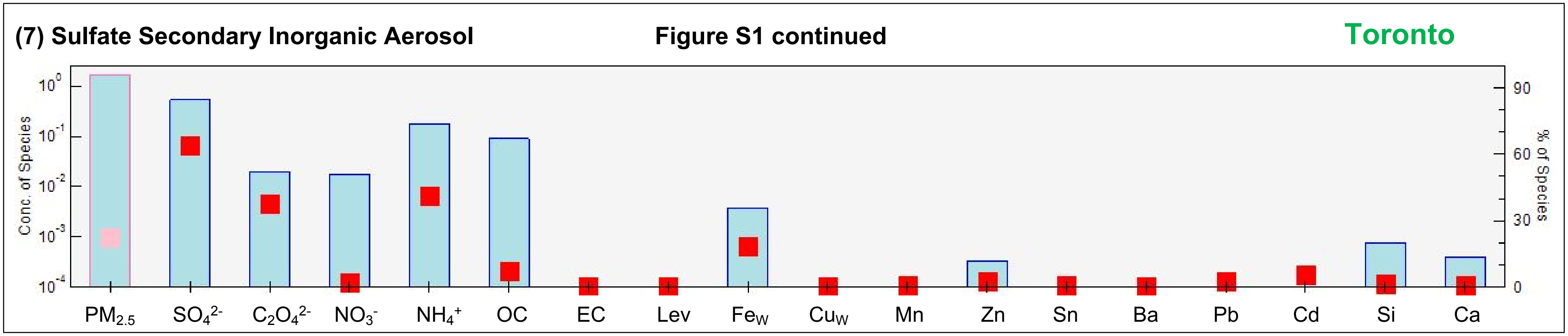

Figure S1: Factor contributions from the PMF analysis of Toronto data**.** Blue bars are the concentrations of species ($\mu g\ m^{-3}$) and red squares show the percentage of each species in each factor. “Lev” denotes levoglucosan.

Table S2. Factor profiles (% of species) from PMF analysis of Toronto data

	Biomass burning	Road dust	Aged carbonaceous aerosol	Industry	Nitrate SIA	Traffic	Sulfate SIA [a]
$PM_{2.5}$	19	0	19	10	13	16	23
SO_4^{2-}	13	0	5	1	10	8	64
$C_2O_4^{2-}$	0	3	56	3	0	0	37
NO_3^-	2	1	0	0	91	4	2
NH_4^+	5	0	0	0	47	6	41
**OC**	39	4	38	2	3	5	7
**EC**	28	0	34	0	3	35	0
**Lev** [b]	59	5	2	4	30	0	0
Fe_w [c]	0	2	61	1	0	18	18
Cu_w [c]	23	1	32	0	0	44	0
**Mn**	0	30	1	25	1	41	1
**Zn**	4	21	1	46	0	26	2
**Sn**	1	32	1	24	1	41	1
**Ba**	0	36	0	2	1	61	0
**Pb**	0	0	1	83	1	12	3
**Cd**	6	4	2	73	0	9	6
**Si**	5	79	3	0	0	12	2
**Ca**	1	37	1	17	1	43	1

[a] SIA: secondary inorganic aerosol; [b] Lev: levoglucosan; [c] subscript "w" denotes water-soluble form.

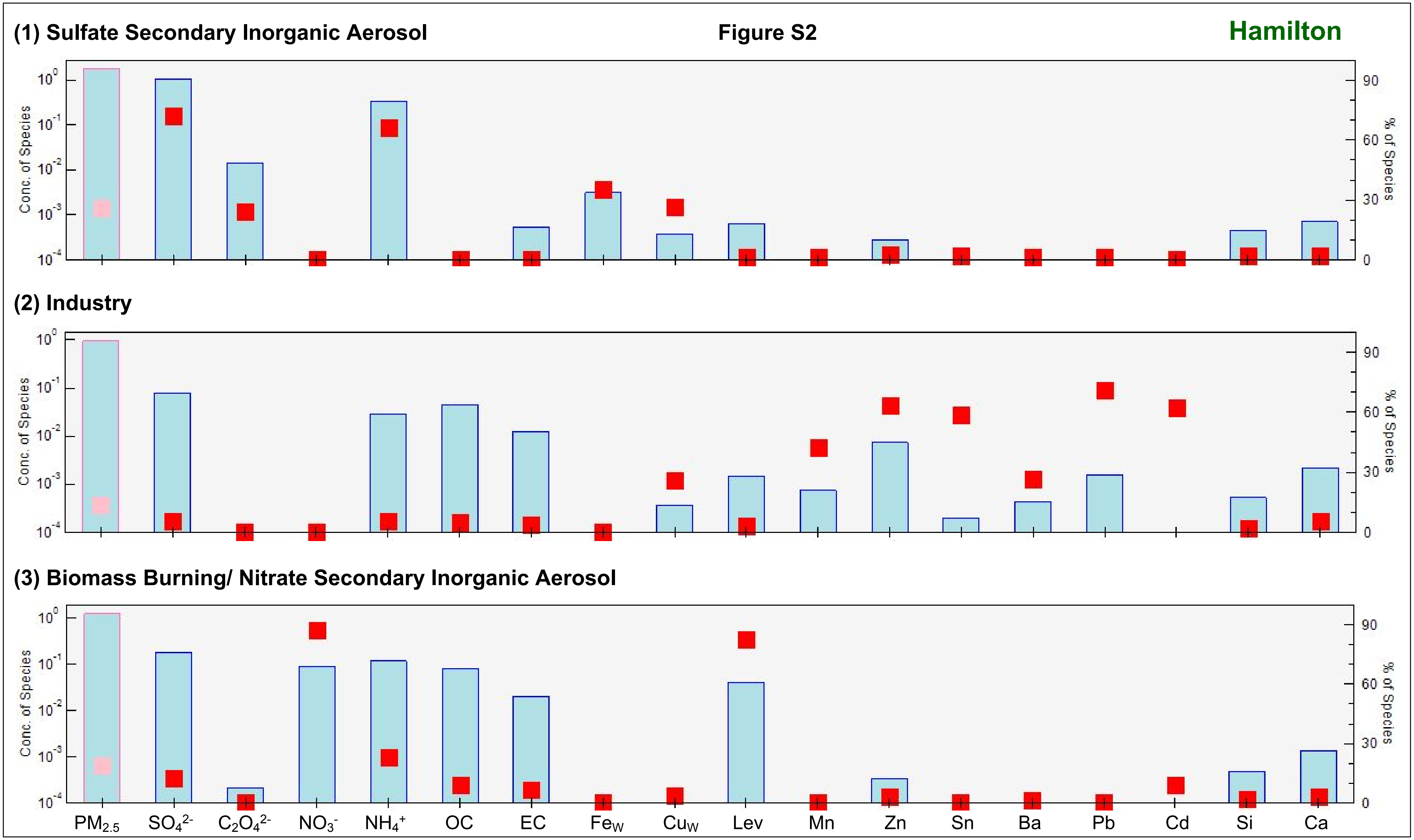
(1) Sulfate Secondary Inorganic Aerosol
Figure S2
Hamilton
(2) Industry
(3) Biomass Burning/ Nitrate Secondary Inorganic Aerosol
Conc. of Species
% of Species
PM2.5
SO4 2-
C2O4 2-
NO3 -
NH4 +
OC
EC
FeW
CuW
Lev
Mn
Zn
Sn
Ba
Pb
Cd
Si
Ca

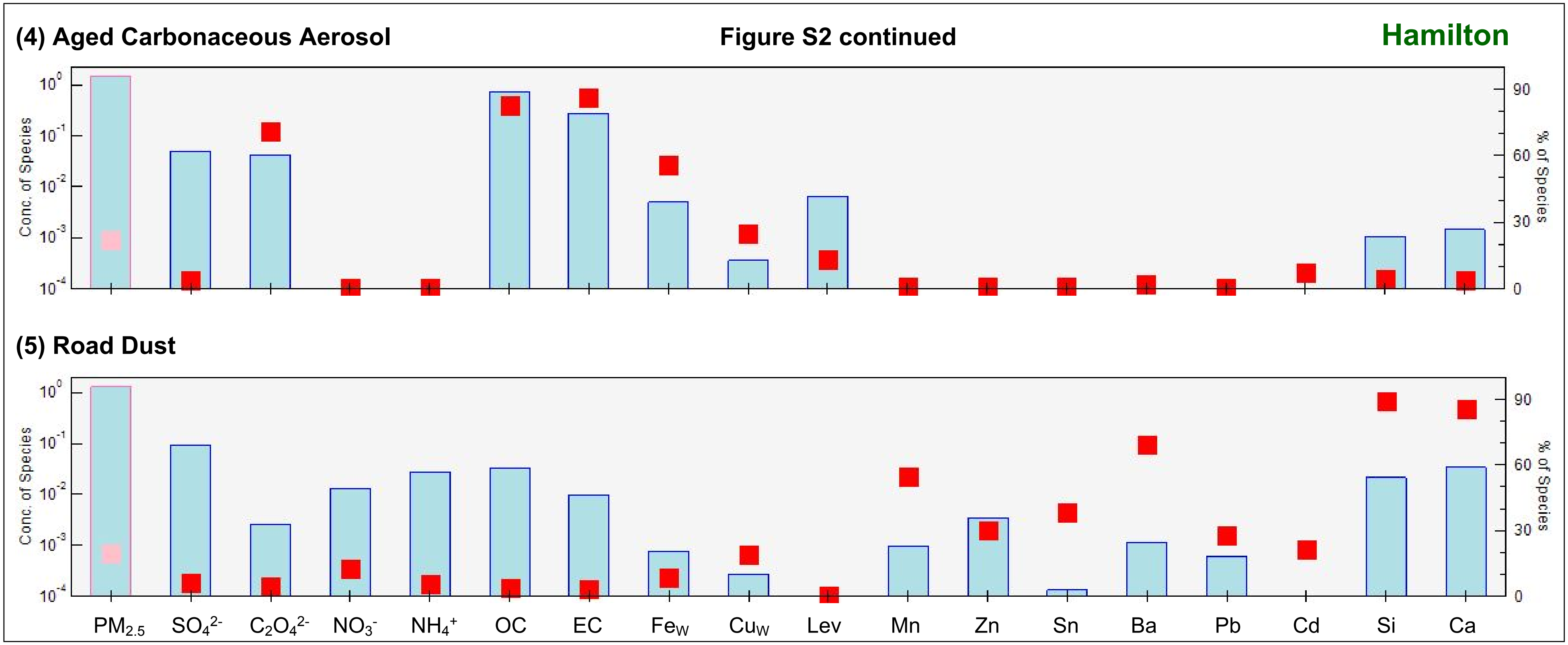

Figure S2: Factor contributions from the PMF analysis of Hamilton data. Blue bars are the concentrations of species (μg m^{-3}) and red squares show the percentage of each species in each factor. "Lev" denotes levoglucosan.

Table S3. Factor profiles (% of species) from PMF analysis of Hamilton data

	Sulfate SIA [a]	Industry	Biomass burning/nitrate SIA	Aged carbonaceous aerosol	Road dust
**$PM_{2.5}$**	26	14	19	22	19
**SO_4^{2-}**	72	5	13	4	6
**$C_2O_4^{2-}$**	24	0	0	71	4
**NO_3^-**	0	0	88	0	12
**NH_4^+**	66	6	23	0	5
**OC**	0	5	9	83	4
**EC**	0	4	6	86	3
**Fe_w** [b]	35	0	0	56	9
**Cu_w** [b]	26	26	4	25	19
**Lev** [c]	1	3	83	13	0
**Mn**	1	42	1	1	55
**Zn**	2	64	3	1	30
**Sn**	2	59	0	1	38
**Ba**	1	26	1	2	69
**Pb**	1	71	0	0	28
**Cd**	0	62	9	7	21
**Si**	2	2	2	4	89
**Ca**	2	5	3	4	86

[a] SIA: secondary inorganic aerosol; [b] subscript "w" denotes water-soluble form; [c] Lev: levoglucosan.

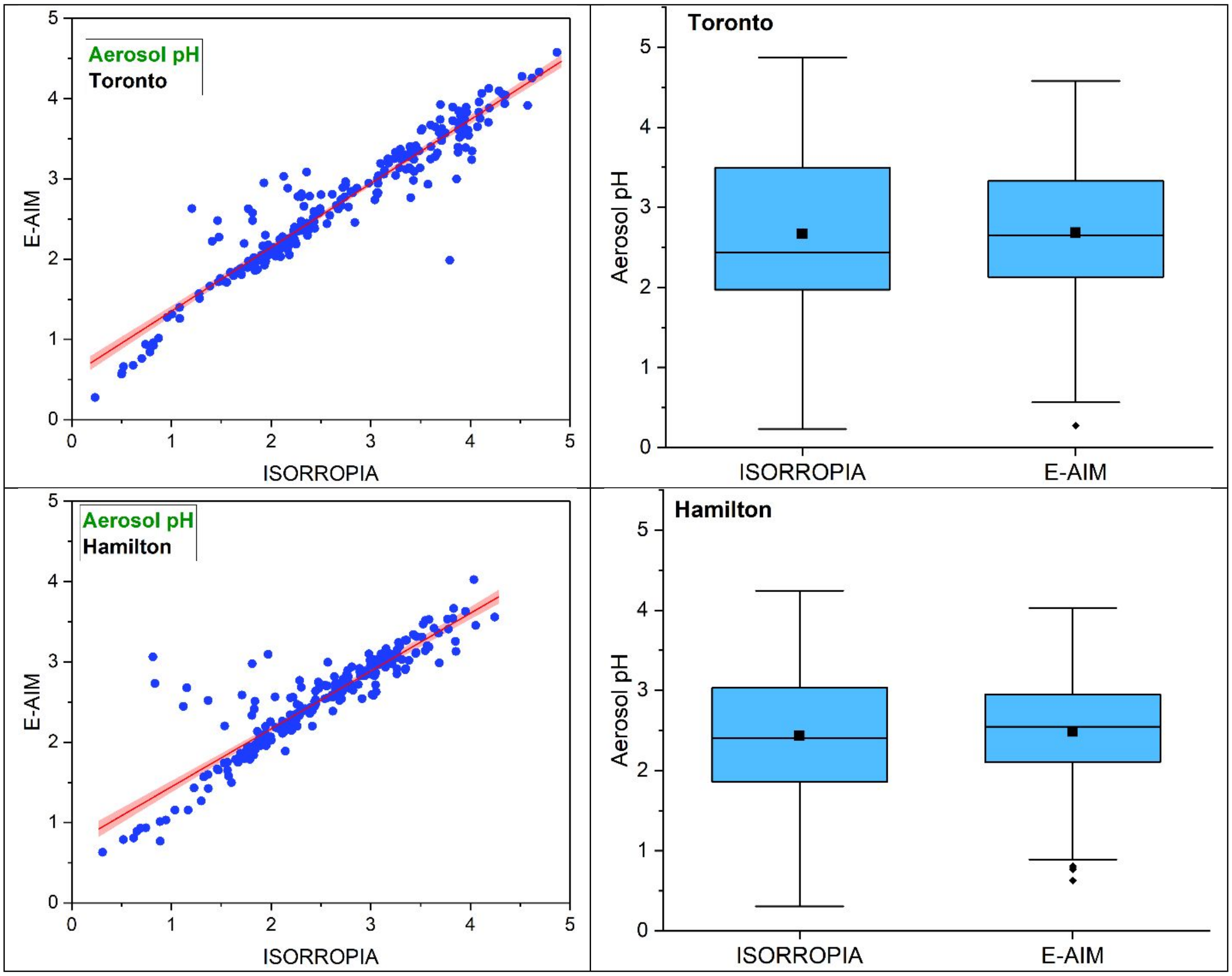

Figure S3. comparison of aerosol pH obtained from ISORROPIA and E-AIM models. The calculations included data for NH_4^+, SO_4^{2-}, NO_3^-, and NH_3, and HNO_3.

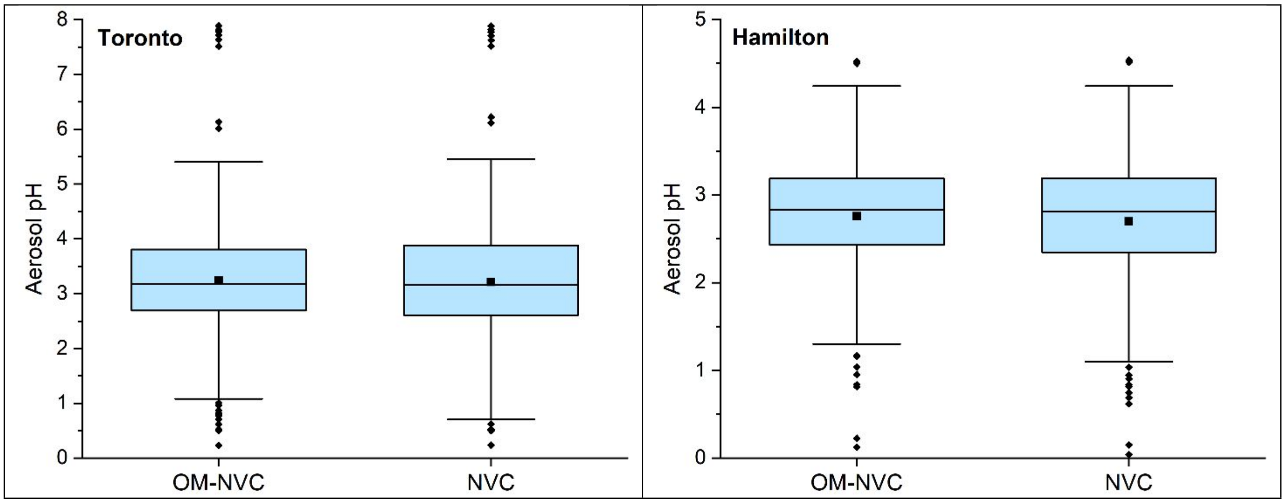

Figure S4. aerosol pH obtained using ISORROPIA model. The analysis included data for NH_4^+, SO_4^{2-}, NO_3^-, NH_3 and and HNO_3, as well as organic matter and non-volatile cations Na, Ca, Mg, and K (i.e. OM-NVC) or non-volatile cations only (i.e. NVC).

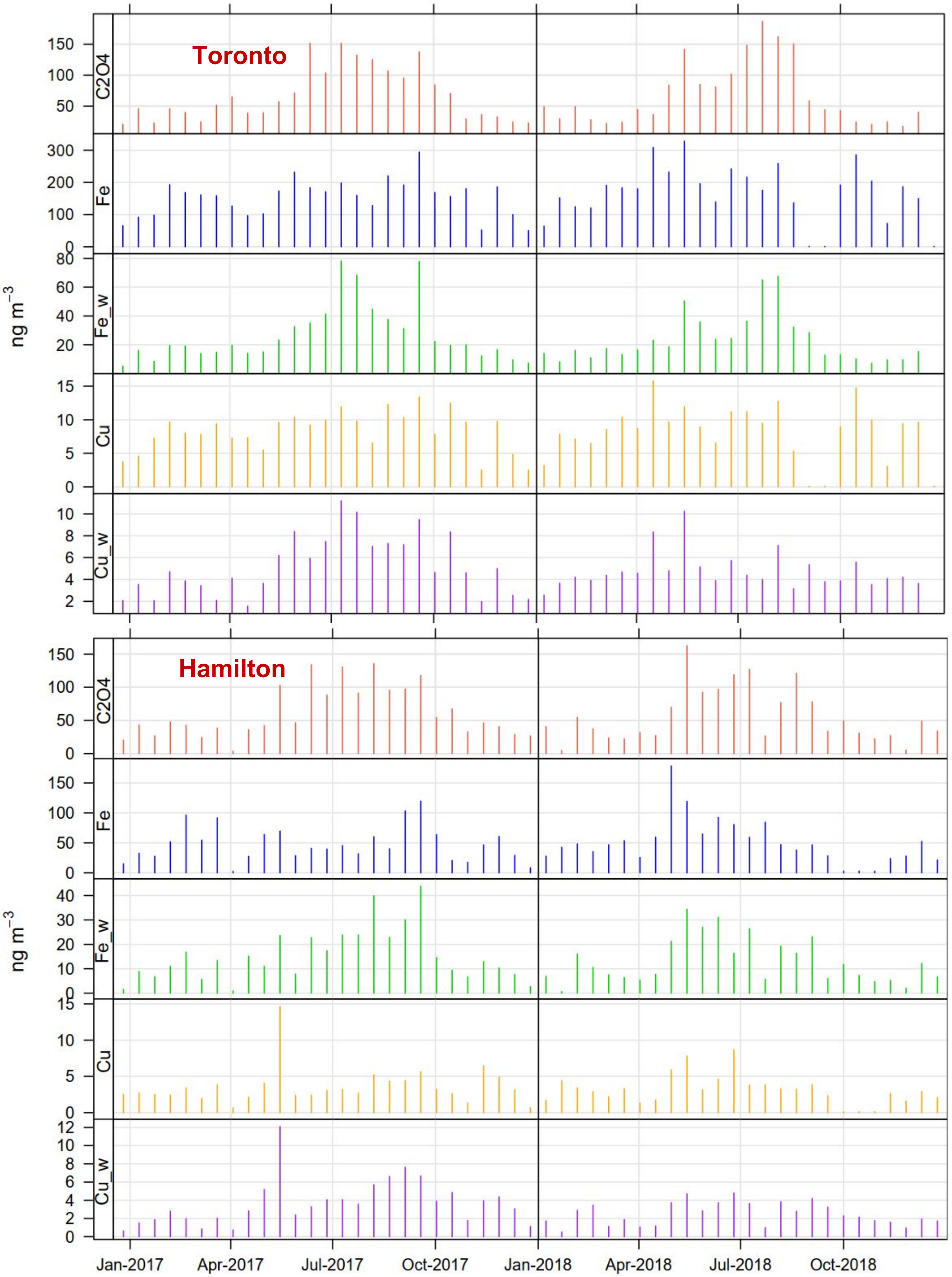

Figure S5a. Biweekly mean concentrations (ng m^{-3}) of Cu, Fe, and their water-soluble forms Cu_w and Fe_w, and oxalate at study sites in 2017-2018 period.

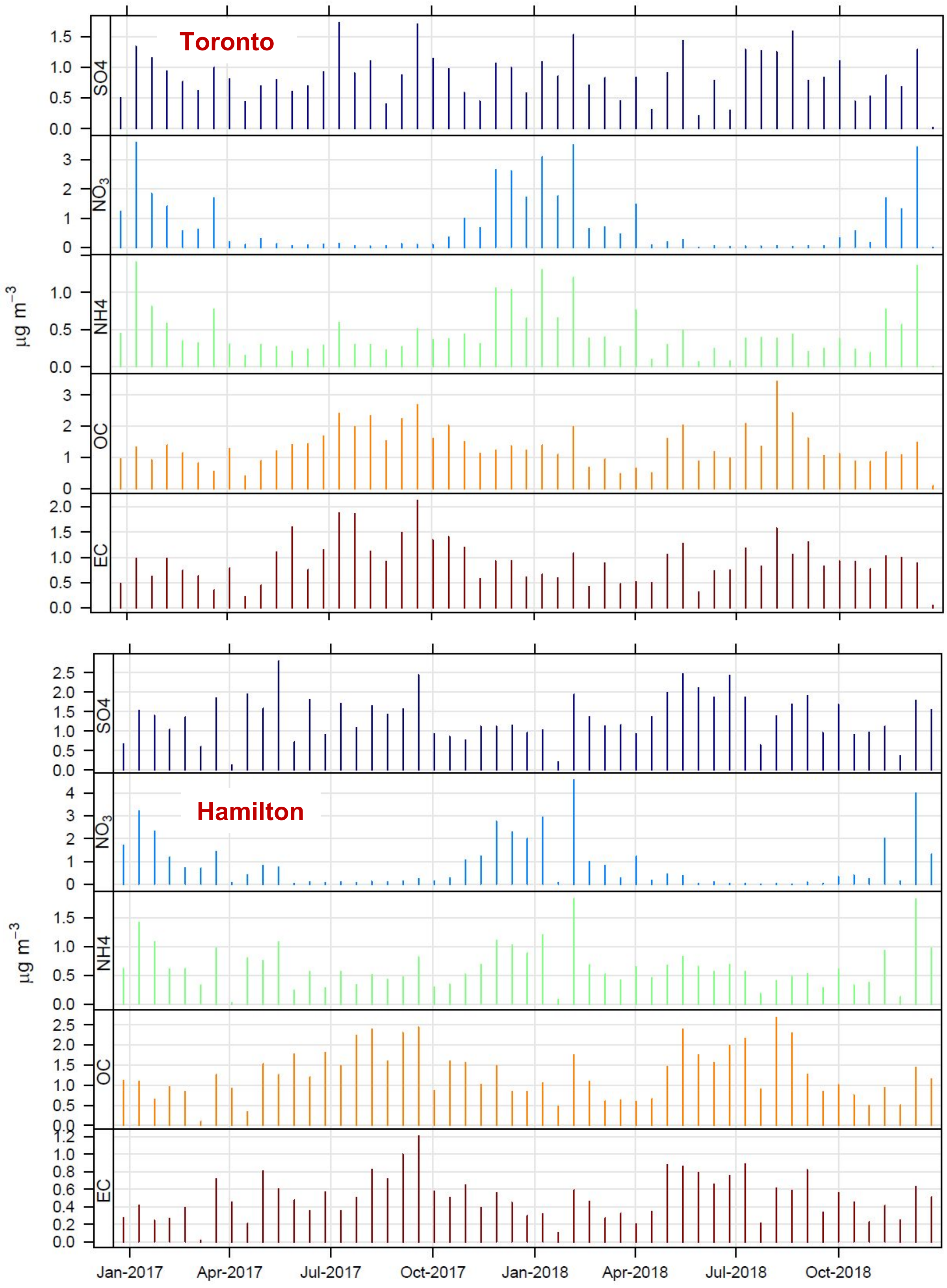

Figure S5b. Biweekly mean concentrations (µg m-3) of sulfate, nitrate, ammonium, organic and elemental carbon at study sites in 2017-2018 period.

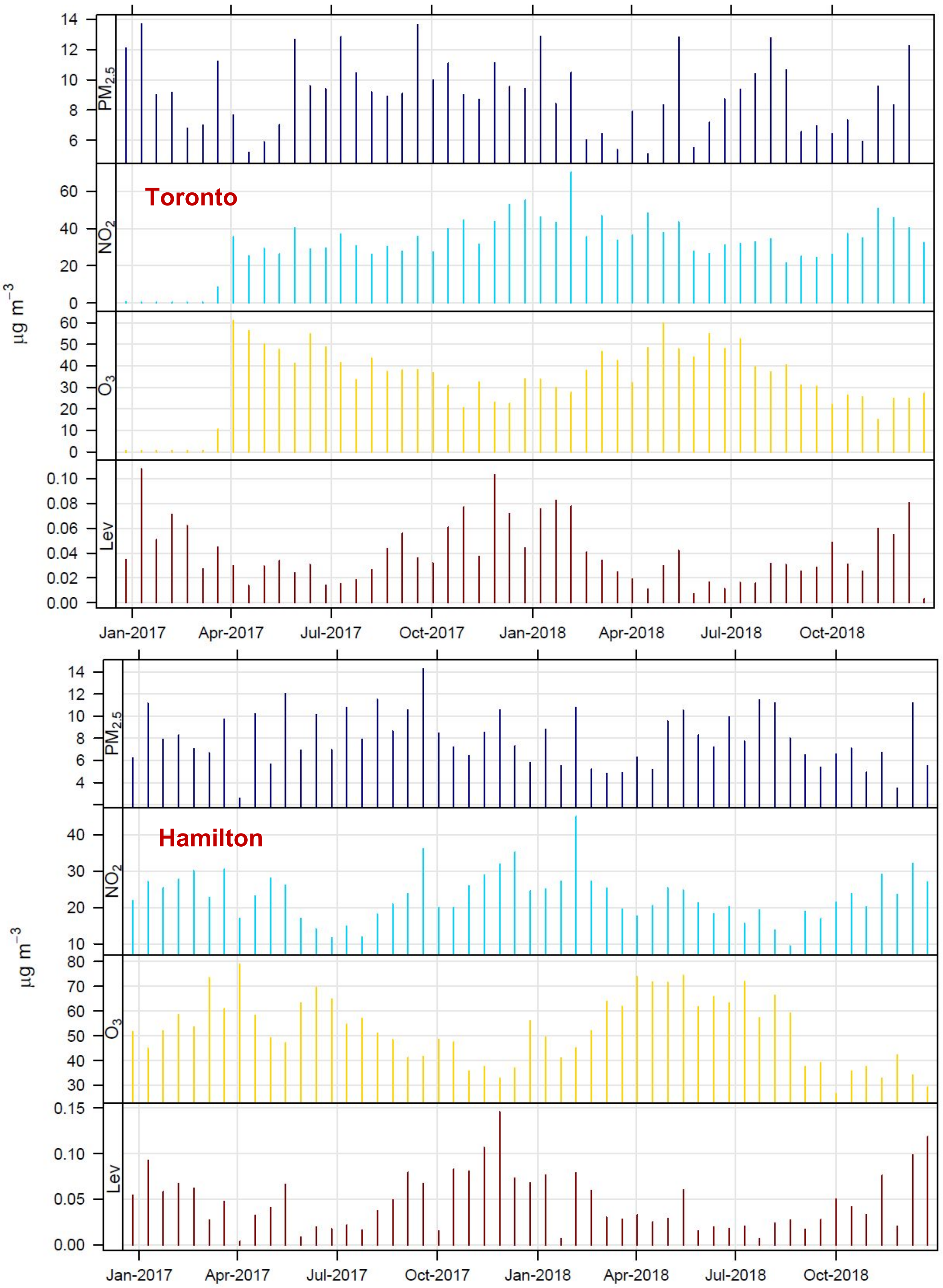

Figure S5c. Biweekly mean concentrations (µg m-3) of PM2.5, levoglucosan (Lev), nitrogen dioxide (NO2), and ozone (O3) at study sites in 2017-2018. Note. NO_2 and O_3 were not available in Jan-Mar, 2017.

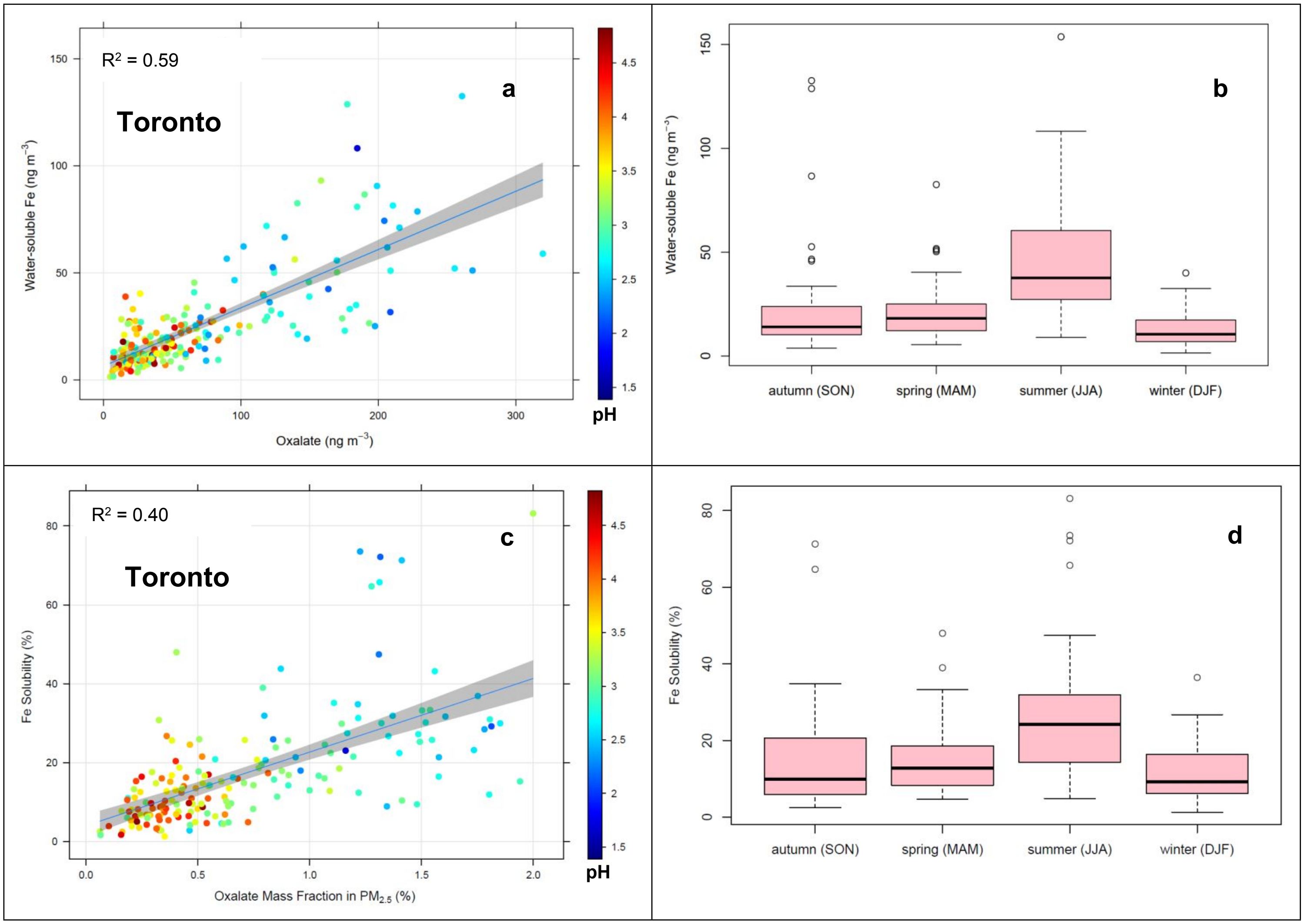

Figure S6. Seasonal variation in oxalate and water-soluble Fe concentrations (ng m^{-3}), Fe solubility, oxalate mass fraction, and aerosol pH at Toronto site. Fe solubility is defined as the ratio of water-soluble to near-total Fe concentrations.

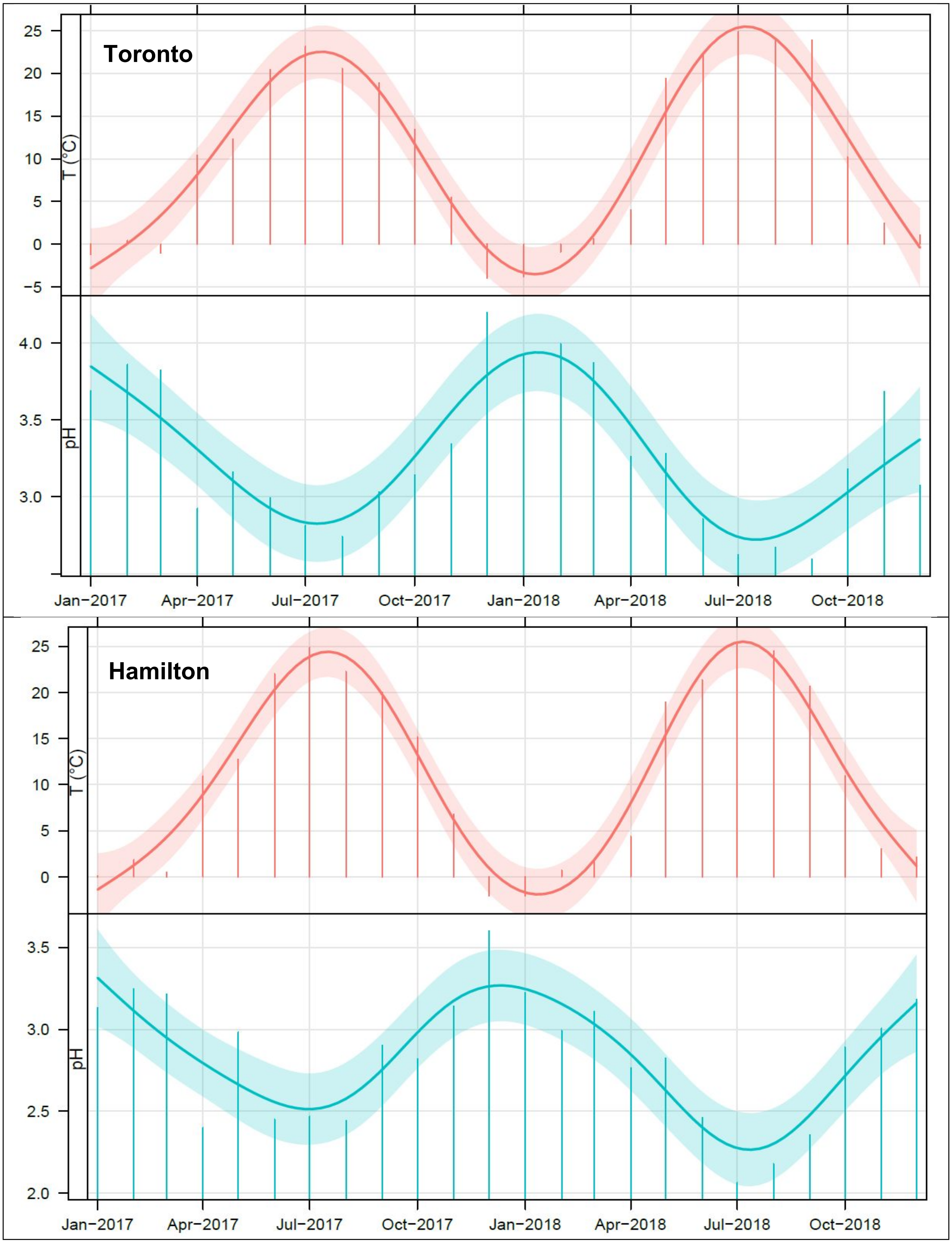

Figure S7. Seasonal changes in ambient temperature (T °C) and aerosol pH and at the study sites in 2017-2018 period. The shading shows the estimated 95% confidence interval for the smooth trend.

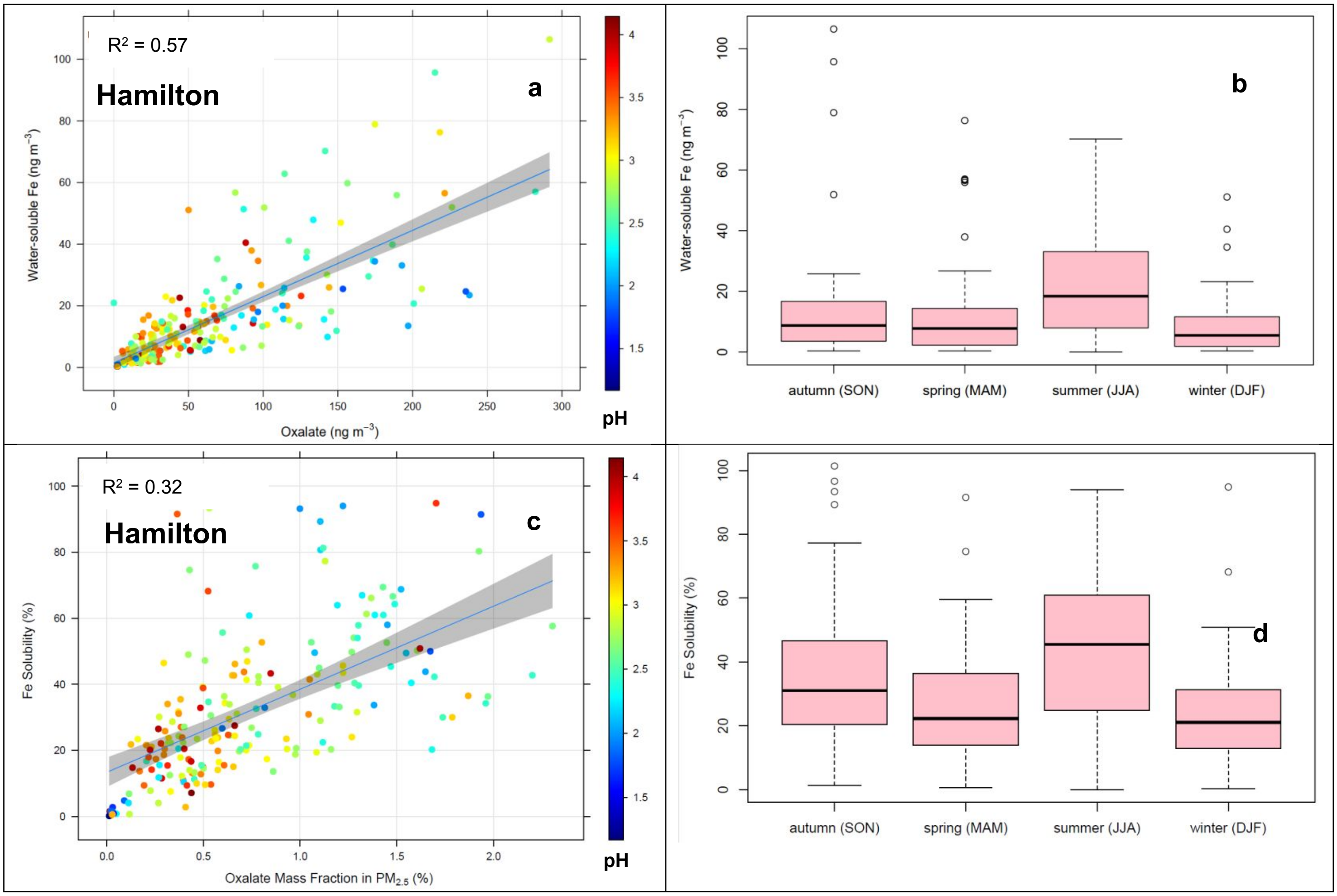

Figure S8. Seasonal variation in oxalate and water-soluble Fe concentrations (ng m^{-3}), Fe solubility, oxalate mass fraction, and aerosol pH at Hamilton site. Fe solubility is defined as the ratio of water-soluble to near-total Fe concentrations.

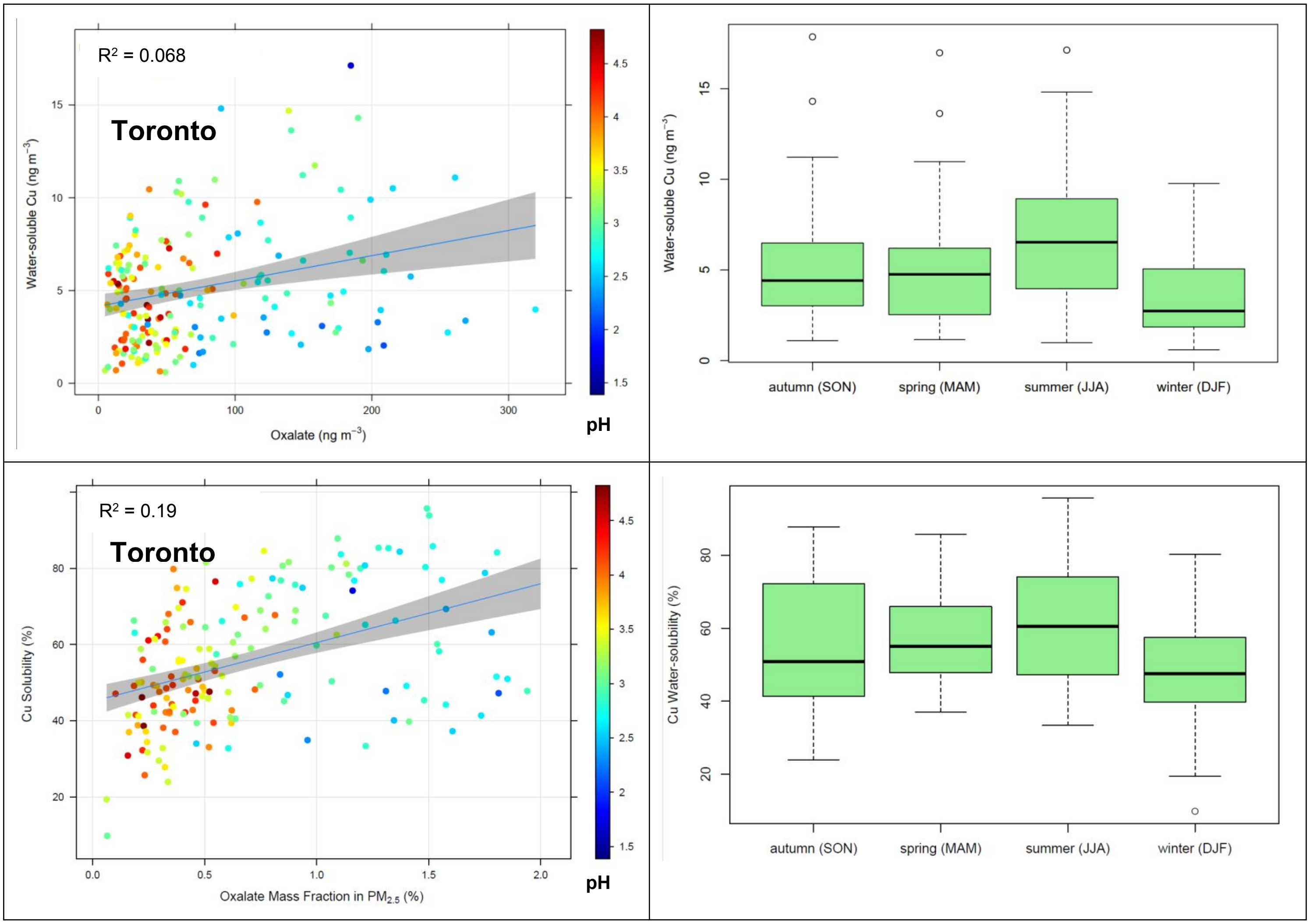

Figure S9. Seasonal variation in oxalate and water-soluble Cu concentrations (ng m^{-3}), Cu solubility, oxalate mass fraction, and aerosol pH at Toronto site. Cu solubility is defined as the ratio of water-soluble to near-total Cu concentrations.

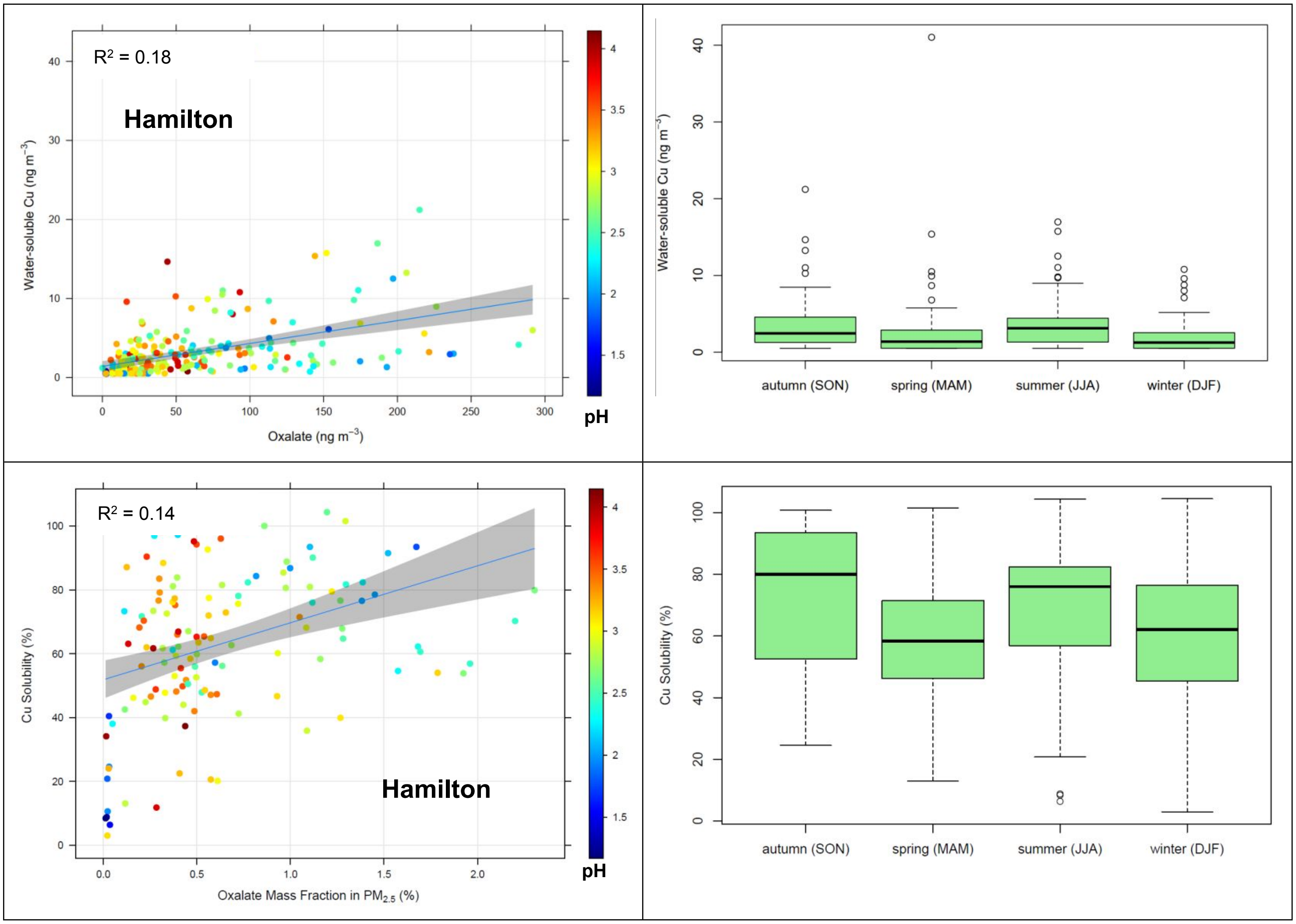

Figure S10. Seasonal variation in oxalate and water-soluble Cu concentrations (ng m^{-3}), Cu solubility, oxalate mass fraction, and aerosol pH at Hamilton site. Cu solubility is defined as the ratio of water-soluble to near-total Cu concentrations.

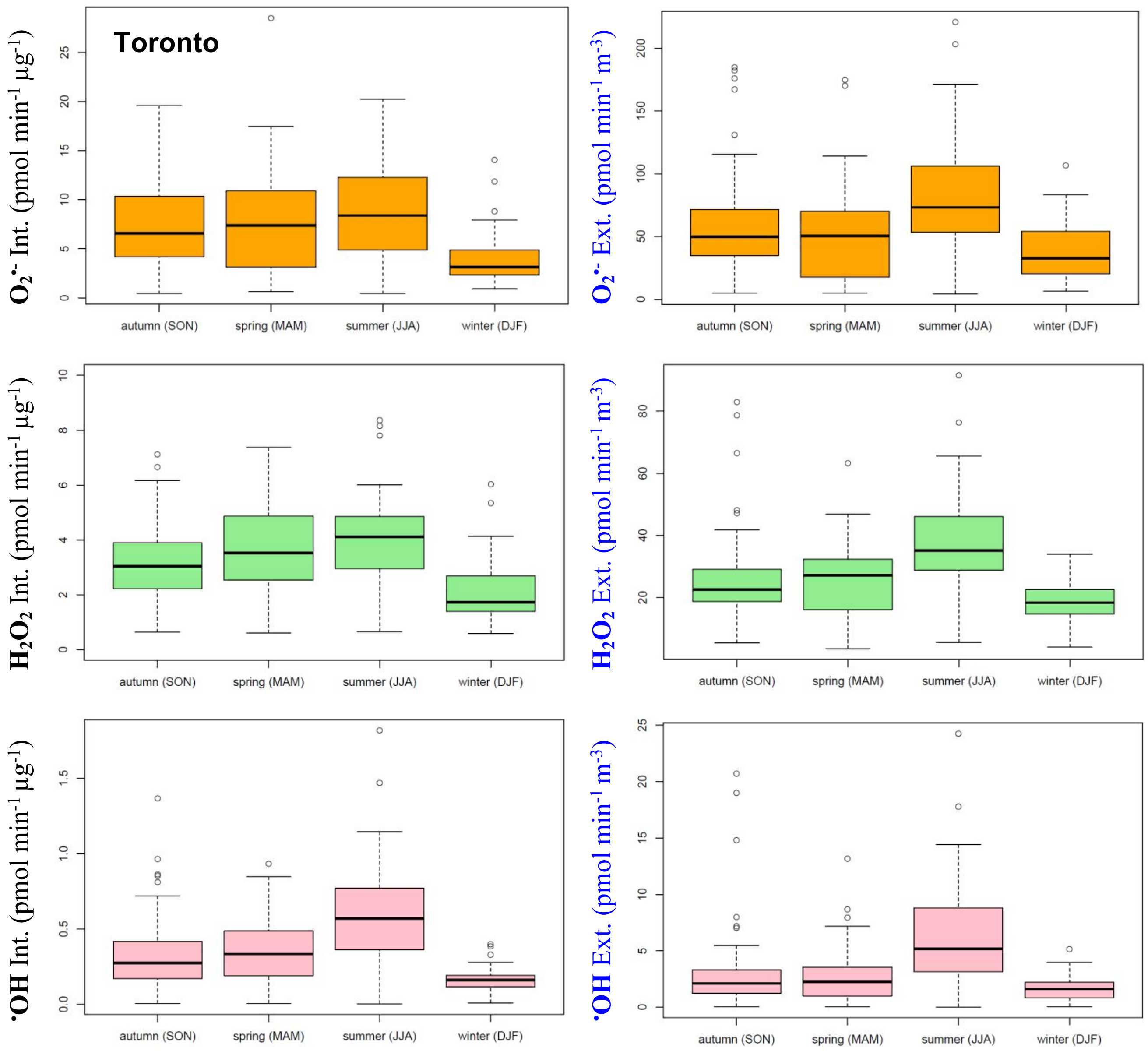

Figure S11. Intrinsic (Int.) and extrinsic (Ext.) ROS production in the lung from the inhalation of ambient air at Toronto site.

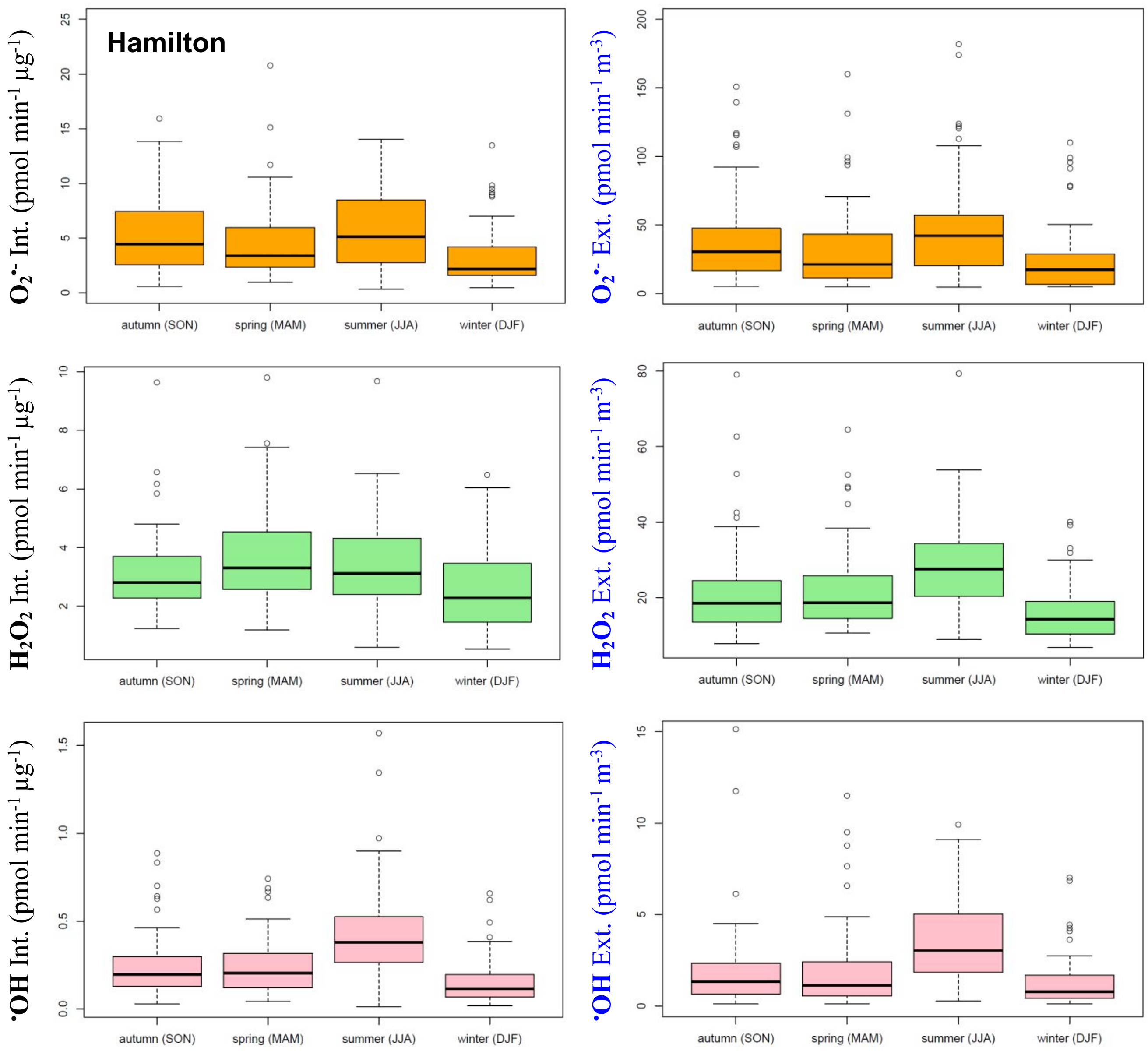

Figure S12. Intrinsic (Int.) and extrinsic (Ext.) ROS production in the lung from the inhalation of ambient air at Hamilton site.

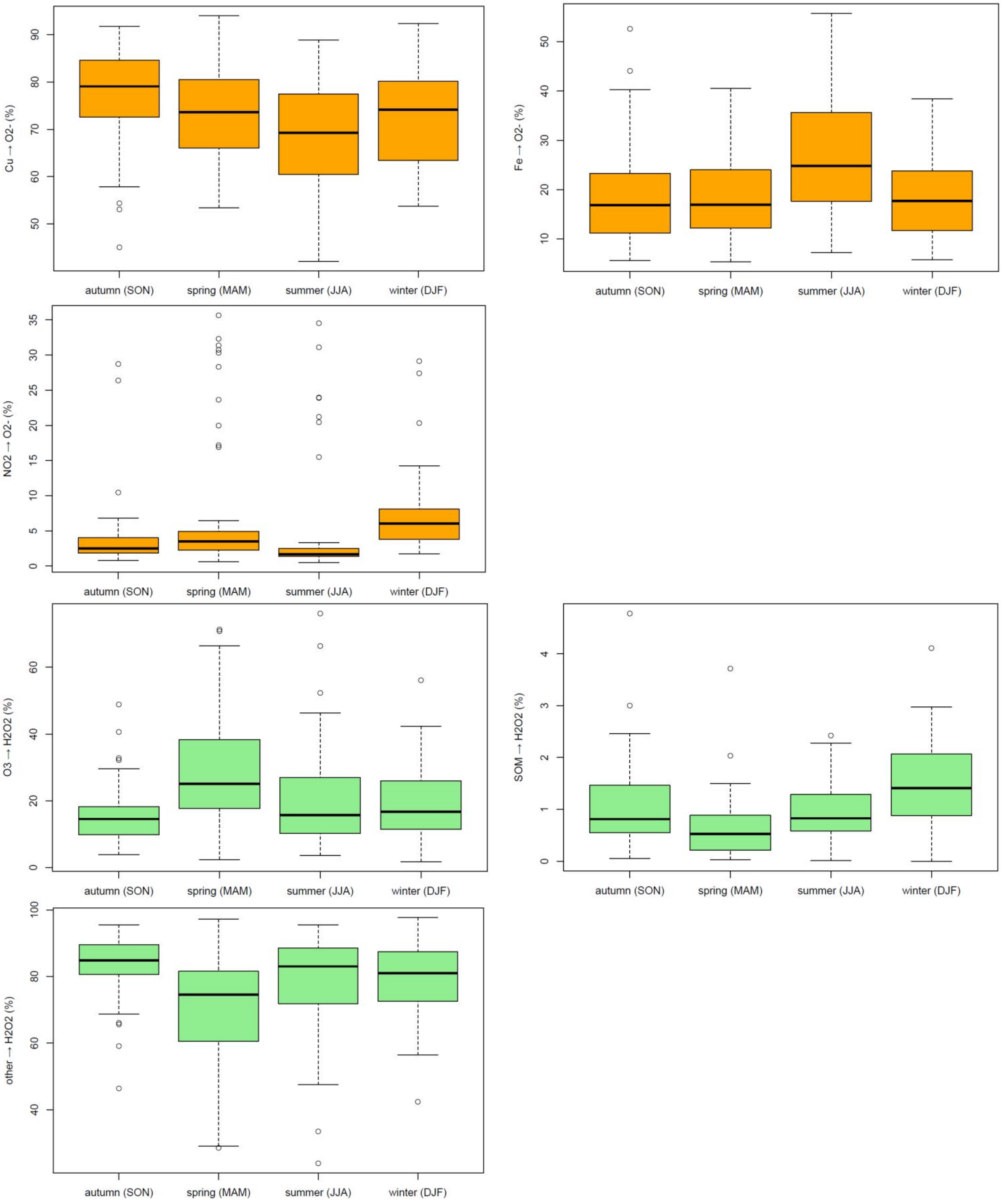

Figure S13. Contribution of various chemical species to ROS formation at **Toronto** site. “Other” denotes the conversion of $O_2^{\bullet -}$ to H_2O_2 through antioxidants and enzymes.

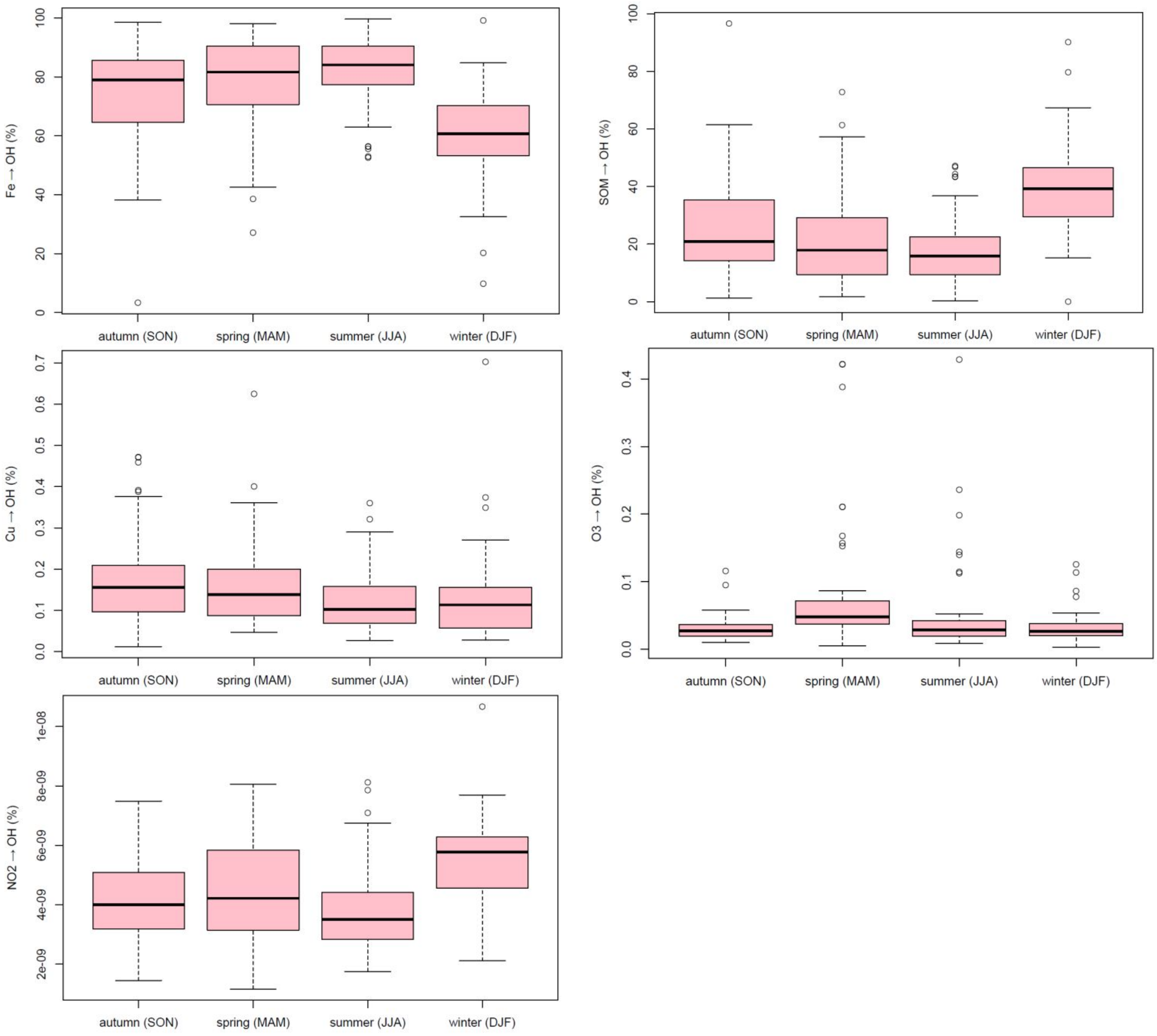

Figure S13 continued. Contribution of various chemical species to ROS formation at Toronto site.

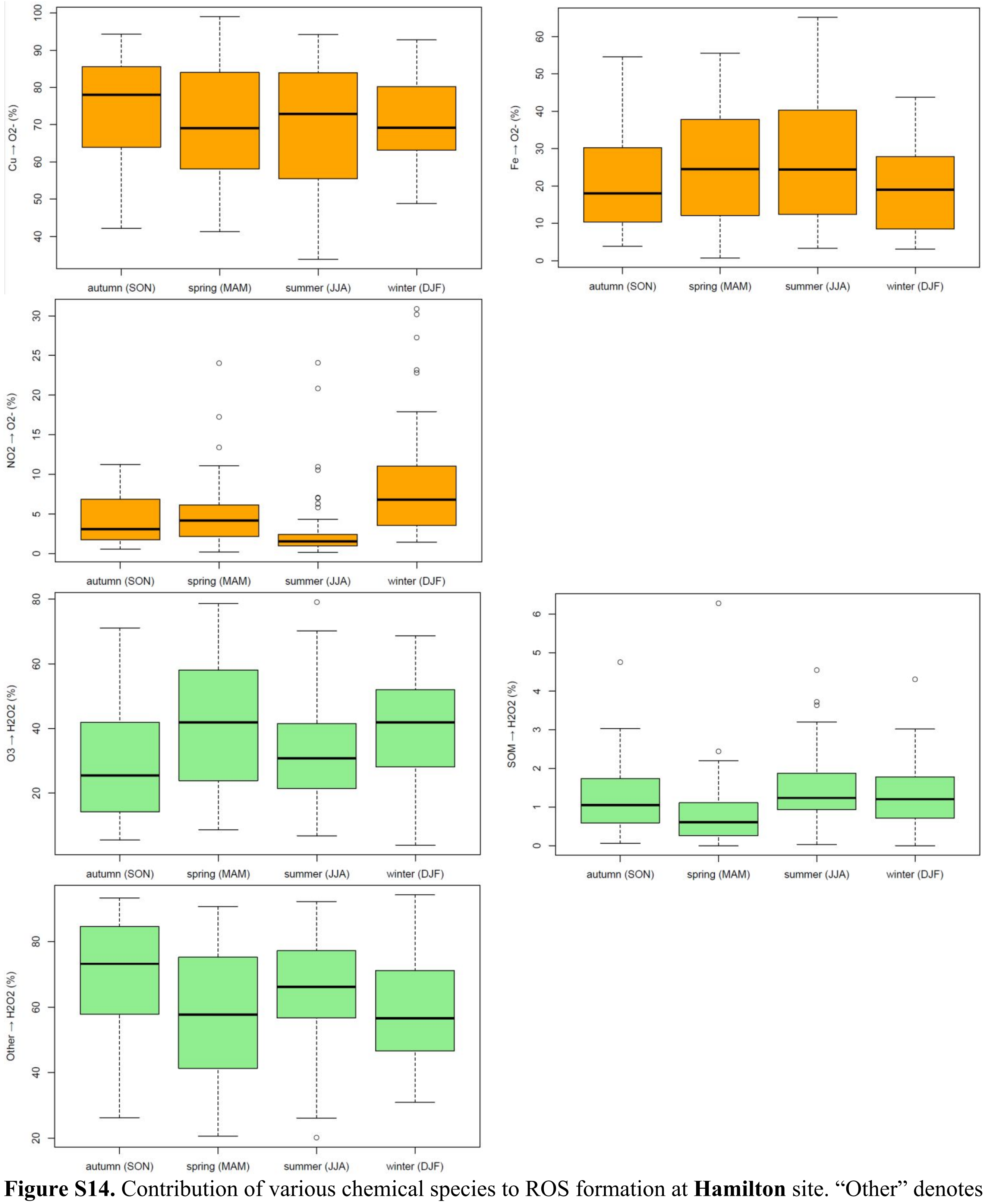

Figure S14. Contribution of various chemical species to ROS formation at **Hamilton** site. "Other" denotes the conversion of $O_2^{\bullet-}$ to H_2O_2 through antioxidants and enzymes.

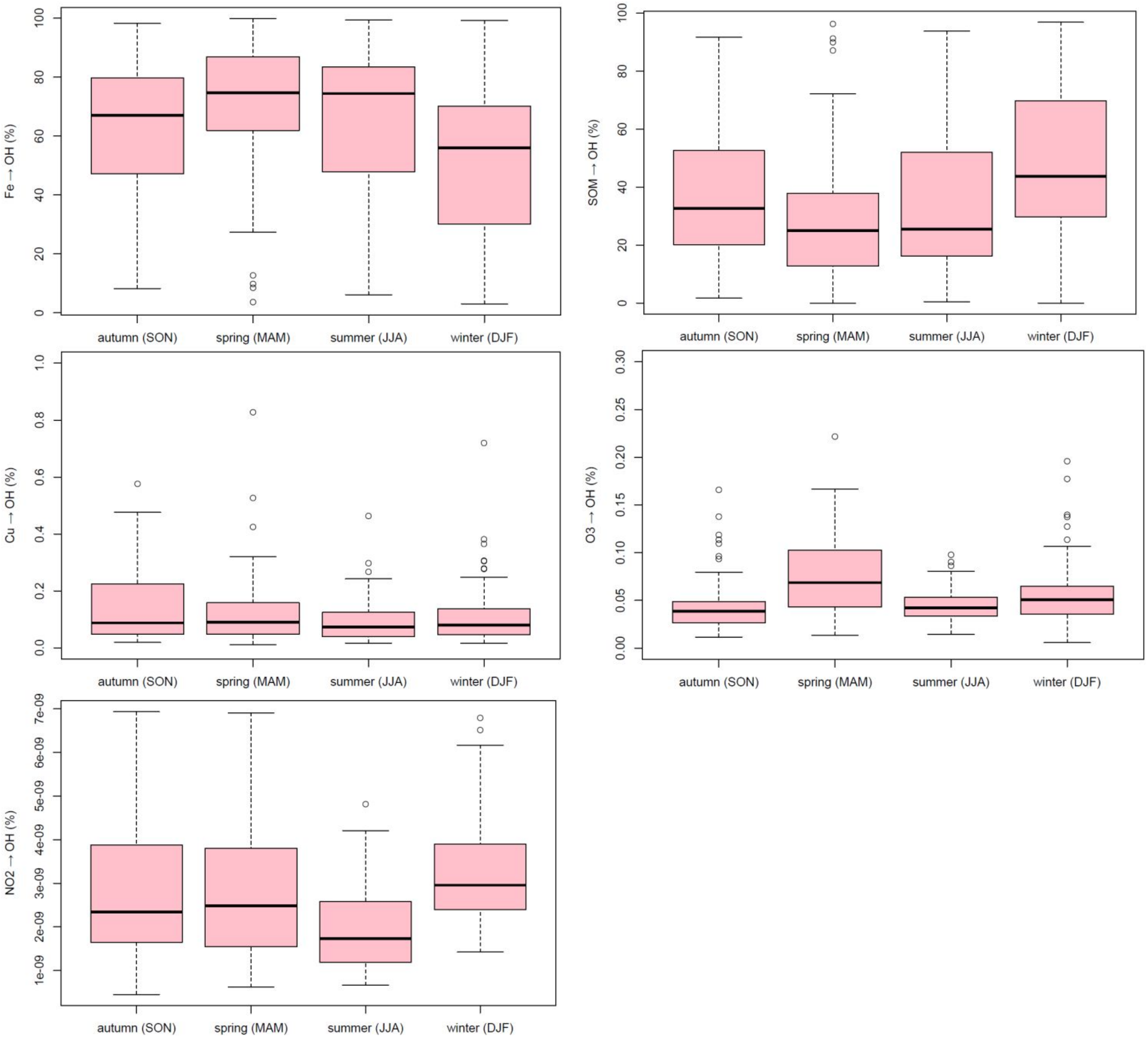

Figure S14 continued. Contribution of various chemical species to ROS formation at Hamilton site.

References

(1) Pye, H. O. T.; Nenes, A.; Alexander, B.; Ault, A. P.; Barth, M. C.; Clegg, S. L.; Collett Jr., J. L.; Fahey, K. M.; Hennigan, C. J.; Herrmann, H.; et al. The Acidity of Atmospheric Particles and Clouds. *Atmos. Chem. Phys.* **2020**, *20*, 4809–4888. https://doi.org/10.5194/acp-20-4809-2020.

(2) Guo, H.; Xu, L.; Bougiatioti, A.; Cerully, K. M.; Capps, S. L.; Hite, J. R.; Carlton, A. G.; Lee, S.-H.; Bergin, M. H.; Ng, N. L.; et al. Fine-Particle Water and pH in the Southeastern United States. *Atmos. Chem. Phys.* **2015**, *15*, 5211–5228. https://doi.org/10.5194/acp-15-5211-2015.

(3) Hennigan, C. J.; Izumi, J.; Sullivan, A. P.; Weber, R. J.; Nenes, A. A Critical Evaluation of Proxy Methods Used to Estimate the Acidity of Atmospheric Particles. *Atmos. Chem. Phys.* **2015**, *15*, 2775–2790. https://doi.org/10.5194/acp-15-2775-2015.

(4) Clegg, S. L.; Brimblecombe, P.; Wexler, A. S. Thermodynamic Model of the System H+ - NH4+ - SO42− - NO3− H2O at Tropospheric Temperatures. *J. Phys. Chem. A* **1998**, *102*, 2137–2154. https://doi.org/10.1021/jp973042r.

(5) Kakavas, S.; Pandis, S. N.; Nenes, A. ISORROPIA-Lite: A Comprehensive Atmospheric Aerosol Thermodynamics Module for Earth System Models. *Tellus B Chem. Phys. Meteorol.* **2022**, *74*, 1-23. https://doi.org/10.16993/tellusb.33.

(6) Nenes, A.; Pandis, S. N.; Pilinis, C. Continued Development and Testing of a New Thermodynamic Aerosol Module for Urban and Regional Air Quality Models. *Atmos. Environ.* **1999,** *33*, 1553-1560. https://doi.org/10.1016/S1352-2310(98)00352-5.

(7) Fountoukis, C.; Nenes, A. ISORROPIA II: A Computationally Efficient Thermodynamic Equilibrium Model for K+ - Ca2+ - Mg2+ - NH4+ - Na+ - SO42− - NO3− - Cl− - H2O aerosols. *Atmos. Chem. Phys.* **2007**, *7*, 4639–4659. https://doi.org/10.5194/acp-7-4639-2007.

(8) Robinson, R. A.; Stokes, R. H. *Electrolyte Solutions*, 2nd Revise.; Dover Publications: New York, 2002.

(9) Tao, Y.; Murphy, J. G. The sensitivity of PM2.5 acidity to meteorological parameters and chemical composition changes: 10-year records from six Canadian monitoring sites. *Atmos. Chem. Phys.* **2019**, *19*, 9309–9320. https://doi.org/10.5194/acp-19-9309-2019.
